# Physical activity interventions for cardiometabolic and psychosocial outcomes in people living with HIV in sub Saharan Africa: A systematic review

**DOI:** 10.1371/journal.pgph.0007070

**Published:** 2026-09-28

**Authors:** Smart Z. Mabweazara, Jennifer Manne-Goehler, Mark Hamer, Jacqueline Cellini, Takshita Sookan, Lisa K. Micklesfield, Mark J. Siedner

**Affiliations:** 1 Department of Clinical Science, Africa Health Research Institute, KwaZulu Natal, South Africa; 2 Harvard Medical School, Harvard University, Cambridge, Massachusetts, United States of America; 3 Brigham and Women’s Hospital, Boston, Massachusetts, United States of America; 4 Division of Surgery and Interventional Science, Institute of Sport Exercise and Health, University College London, London, United Kingdom; 5 Department of Biokinetics, Exercise and Leisure Sciences, College of Health Sciences, University of KwaZulu Natal, KwaZulu Natal, South Africa; 6 Massachusetts General Hospital, Boston, Massachusetts, United States of America; 7 Department of Paediatrics, South African Medical Research Council/Wits Developmental Pathways for Health Research Unit, University of the Witwatersrand, Johannesburg, South Africa; Indiana University Richard M Fairbanks School of Public Health, UNITED STATES OF AMERICA

## Abstract

Antiretroviral therapy (ART) has extended the lifespan of people living with HIV (PWH), resulting in a growing burden of cardiometabolic disease (CMD) associated with chronic HIV infection and the effects of ART. Physical activity (PA) may reduce CMD risk while improving psychosocial health among PWH. We systematically reviewed the efficacy of PA interventions for improving cardiometabolic and psychosocial outcomes among PWH in sub-Saharan Africa (sSA). Web of Science, PubMed, African Index Medicus, Embase, and CINAHL were searched from inception to 10 August 2023 for randomised controlled trials and quasi-experimental studies published from 2000 onwards involving adults with HIV in sSA. Methodological quality was assessed using the PEDro scale and Downs and Black checklist, and findings were synthesised narratively. Seventeen studies involving 1,130 participants met the inclusion criteria and generally demonstrated good methodological quality. Most participants were female (51%), and all studies included PWH receiving ART. Interventions included aerobic exercise (n = 8), resistance exercise (n = 3), combined aerobic and resistance exercise (n = 5), while one study did not report exercise modality. Intervention durations ranged from 6 weeks to 12 months. Nearly all studies reported improvements in cardiometabolic outcomes, including blood pressure, lipids, body mass index, body fat percentage, and waist circumference, and/or psychosocial outcomes, including depression, anxiety, self-efficacy and quality of life. Two studies assessing PA outcomes reported significant improvements in step count and metabolic equivalents per week. Limitations included small sample sizes and generally short post-intervention follow-up, limiting assessment of sustained benefits and cardiovascular events. PA interventions may therefore be a valuable addition to standard HIV care in sSA. Future research should identify optimal exercise modalities, duration, and intensity and develop feasible, context-sensitive, scalable interventions, particularly for resource-constrained and underserved populations.

**Systematic review registration:**

PROSPERO registration ID: CRD42021271937

## Introduction

Of the 37 million people living with HIV (PWH), approximately 68% live in sub-Saharan Africa (sSA) [1]. Health priorities for PWH in sSA have significantly transformed in the region, with the worldwide dissemination of antiretroviral therapy (ART) [2]. Widespread availability of ART has transformed HIV from a frequently fatal condition into a manageable chronic disease, extending life expectancy to nearly that of uninfected individuals when started early and maintained [3,4]. As life expectancy has increased, there has been a higher incidence of non-communicable diseases within this population [5]. Cardiovascular and metabolic diseases (CMDs) have been identified as the most common and most severe non-communicable diseases among PWH [6] and multimorbidity is already high in sSA populations [7]. For example, the Randomized Trial to Prevent Vascular Events in HIV (REPRIEVE) study recently reported a median BMI of 25.9 kg/m^2^ and that 36% of the 7382 participants had hypertension [8]. These conditions are of particular concern given the use of newer ART regimens, such as dolutegravir, which are strongly linked to obesity, particularly in sSA [9].

Physical activity (PA) has proven to be effective for reducing the likelihood of CMD in PWH [10]. Resistance exercise, for example, is linked to significant reductions in high-density lipoprotein, total cholesterol, low-density lipoprotein, and triglycerides [11]. Resistance exercise is also associated with significant reductions in haemoglobin A1c (HbA1c), fasting blood glucose, body fat percentage [12], and blood pressure [13], and increased lean body mass percentage in PWH [10,14]. Physical activity has also been found to increase cardiorespiratory fitness [15]. There is also overwhelming evidence reported in systematic reviews for the efficacy of PA to improve body composition [16,17], fitness and functional capacity [18,19], and some psychosocial conditions, including quality of life and mental health [20,21] among PWH. These benefits of PA underscore the necessity of promoting its implementation for PWH, particularly in regions where public health systems encounter significant resource limitations [2]. As CMDs increasingly contribute to illness and death among PWH and given the established link between PA and enhanced CMD outcomes in various populations, comprehensively grasping methods to promote PA among individuals with HIV in sSA will be an essential initial stride [2]. This will aid in addressing an unfulfilled health requirement for this population [2].

Given the differences in HIV/AIDS burden, access to healthcare, and socioeconomic factors between regions, particularly the Global North and South, coupled with variations in PA interventions, a systematic review on the effects of PA among PWH sSA is warranted to provide evidence on its impact in this context. We include studies published after 2000 because the early 2000s marked the beginning of massive ART roll-out, with the number of PWH on treatment in sSA rising from 11,000 in 2000 to over 11 million by 2015 [22]. The development of low-cost generic ART and the launch of major international funding initiatives, specifically the President’s Emergency Plan for AIDS Relief, launched in 2003, caused a massive, rapid expansion in HIV treatment access [23]. The rapid scale-up of ART significantly decreased AIDS-related deaths and improved life expectancy, shifting HIV from a fatal disease to a chronic, manageable condition [24]. As the population of PWH ages, the focus of care has shifted to include managing long-term comorbidities such as cardiovascular disease, diabetes, cancer, and neurocognitive disorders alongside HIV, requiring a strong emphasis on lifestyle interventions [25–27].

We further restricted inclusion to studies conducted in sSA to ensure that findings are contextually relevant and applicable to populations most affected by HIV. Sub-Saharan Africa bears a disproportionate share of the global HIV burden, accounting for nearly two-thirds of all PWH, making it the region where interventions are most urgently needed [28]. In addition, sSA, particularly South Africa, is experiencing a dual burden of disease, characterised by high HIV prevalence alongside rapidly increasing rates of overweight and obesity. In South Africa, more than half of adults are overweight or obese, with prevalence disproportionately higher among women, including those living with HIV [29]. Furthermore, sSA settings are shaped by unique socioeconomic and structural constraints, including poverty, food insecurity, limited access to recreational facilities, and unsafe environments for PA [30,31]. These contextual realities are likely to influence both the design and implementation of PA interventions, meaning that studies conducted in the region may differ in important ways from those undertaken elsewhere, even if similar health-enhancing effects are ultimately observed. To our knowledge, however, no systematic review has specifically examined the efficacy of PA interventions for improving cardiometabolic and psychosocial outcomes among PWH in sSA. As a result, our interpretations and comparisons will necessarily draw largely on evidence from systematic reviews conducted outside the region or not specifically focused on sSA.

In this study, we examined the features and effects of PA interventions aimed at enhancing CMD and psychosocial outcomes in PWH in sSA [2]. A secondary aim was to describe the interventions and identify the behaviour change techniques (BCTs) that were used in these interventions to help inform best practices for elements of such interventions to be used in practice.

## Methods

We adhered to the guidelines of the Preferred Reporting Items for Systematic review and Meta-analysis Protocols (PRISMA-P) [32]. The populated PRISMA checklist for the review is shown in S1 Checklist. A protocol for the systematic review exists and was registered in PROSPERO (I.D: CRD42021271937) [2]. Initially, in our protocol we had planned to use the revised tool to assess the risk of bias in randomised trials (RoB 2) [33]. However, due to a lack of expertise in using RoB 2 within our team, we opted to use the PEDro scale [34] in this review instead. We also intended to assess the certainty of evidence using the Grading of Recommendations Assessment, Development, and Evaluation (GRADE) guidelines [35]. Unfortunately, this was not done because a meta-analysis was not conducted due to the extensive heterogeneity of the interventions included in our review.

### Inclusion criteria

We developed a library search to identify published manuscripts with the following criteria [2]:

1. Publication date in 2000 or after
2. Studies including adults (18 years or older) living with HIV in sSA.
3. Randomized clinical trials or quasi-experimental study designs that evaluated PA-based interventions. No specific comparator or control group was required, but could include passive (e.g., usual care) or active (e.g., alternative behavioural approach) control group.
4. Primary outcome must include at least one cardiometabolic health indicator, such as body composition including BMI, waist circumference, body weight, waist-to-hip-ratio, lipids, measures of diabetes risk, blood pressure and/or a psychosocial outcome/s, or health indicator, such as HRQOL, anxiety, depression, self-efficacy, and social support. Secondary outcomes are PA outcomes and BCTs used in effective PA promotion interventions.

We excluded manuscripts that did not include original data about the effects of PA on cardiometabolic and psychosocial outcomes as well as observational studies, case series, and unpublished pre-prints.

### Sources of information

We conducted systematic searches of five electronic databases: Embase, PubMed, Web of Science, African Index Medicus, and the Cumulative Index to Nursing and Allied Health Literature [2], from 15 May 2021 to 10 August 2023. Articles that were published in any language were included, being translated in Google translator when the language was not native to the authors. Reference lists of previous reviews and included papers were searched to check for any further studies.

We did not search for any grey literature (e.g., reports, theses, conference abstracts, and non–peer-reviewed documents), as we focused exclusively on published randomised controlled trials and quasi-experimental study designs. We excluded grey literature to ensure the inclusion of methodologically rigorous, high-quality evidence. Peer-reviewed publications undergo formal evaluation processes that enhance the validity, reliability, and transparency of study methods and findings, which is critical for synthesising evidence on PA interventions and their cardiometabolic and psychosocial outcomes [36]. Grey literature often lacks detailed reporting of study design, intervention components, outcome measurement, and statistical analyses, limiting the ability to critically appraise methodological quality and risk of bias using established tools [37]. This is particularly important in intervention research, where insufficient reporting can compromise reproducibility and interpretation of effectiveness. While excluding grey literature may increase the risk of publication bias, this limitation is mitigated by conducting comprehensive searches across multiple databases, screening reference lists, and applying transparent inclusion criteria, as recommended by PRISMA guidelines [38]. The search strategy is available in S1 Appendix.

### Summary of manuscript identification

#### Screening.

The full search output was imported into EndNote (Version 20) first, then into Covidence software (Veritas Health Innovation 2020) for screening. The Covidence software assisted in handling the data and study records throughout the review. Two authors (J.M-G and S.Z.M) screened the first 250 abstracts in duplicate, in sets of 50 [2]. In instances where agreement between the 2 authors exceeded Cohen κ > 0.90 for 2 consecutive sets of 50 abstracts, a single reviewer (S.Z.M.) screened the remainder of the abstracts [2]. The same strategy was applied for full text screening.

### Extraction of data

Each included study was analysed using a standardized consensual form to obtain the following data: study year, country, study design, intervention setting, outcomes, and participant characteristics such as age and CD4 count [2]. Intervention features also extracted included: description of the intervention, frequency of the exercise sessions and duration of the intervention, exercise modality, description of the control or comparator intervention, description of primary and secondary outcome measures, and results, by treatment arm [2]. Missing elements of these features were also captured. We used differences in mean from pre-to post-intervention to estimate the effect of the intervention on cardiometabolic outcomes. These were either calculated or extracted from the published manuscripts. Missing or incomplete data were addressed in a stepwise manner. When key outcomes, sample characteristics, or effect estimates were not reported, we contacted corresponding authors via email. If no response was received after two attempts spaced two weeks apart, the study was retained in the review, and the missing data were marked as “NR” (Not Reported).

### Ascertainment of quality of the included studies

We used the PEDro scale [34] to assess the methodological quality of the included randomised controlled trials (RCTs). The PEDro scale scores 11 items: *random allocation, concealed allocation, similarity at baseline, subject blinding, therapist blinding, assessor blinding, > 85% follow up for at least one key outcome, intention-to treat analysis, between-group statistical comparison for at least one key outcome and point and variability measures for at least one key outcome*. Items are scored as either present [1] or absent (0) and a score out of 11 is obtained by summation. An inter-rater reliability generalised kappa statistic of between 0.40 and 0.75 has been reported for the PEDro scale [39]. Studies that did not reach a cut-off of five points [40] were excluded from subsequent analysis.

For non-randomised studies, methodological quality was assessed using the Downs and Black [41] checklist. Methodological quality was assessed using the Modified Downs and Black checklist, which simplifies the original scoring system, particularly the power item, to improve feasibility and inter-rater reliability in systematic reviews including randomised and non-randomised studies [41–43].

In particular, the Modified Downs & Black checklist refines the original 27-item tool to better suit non-randomised study designs, such as cohort and case–control studies, by modifying or de-emphasising items specific to RCTs, particularly those related to randomisation concealment and blinding [41]. Ultimately, this results in a more versatile and balanced instrument for assessing and comparing the methodological quality of both randomised and non-randomised studies in systematic reviews. The item that pertains to the power of the study is modified from a five point score to a one point score which instead assesses whether or not the study performed a power calculation [41]. Thus, the maximum score of the checklist becomes 28 instead of 32.

Downs and Black score ranges were given corresponding quality levels as previously reported [42]: excellent [26–28]; good [20–25]; fair [15–19]; and poor (≤ [14]. Because some questions (items 14, 15, 21–25, 27) are not relevant for uncontrolled studies, the maximum score for uncontrolled studies was 20 points. Thus, for uncontrolled studies we considered: excellent [18–20]; good [12–17]; fair [7–11]; and poor (≤ [6]. Two authors (SZM and JM-G) were tasked with individually assessing the quality of the studies. In cases of disagreement between the reviewers, a third reviewer (MS) was consulted [2]. The ultimate decision regarding the study’s rating was reached through collaborative discussion and consensus among all reviewers.

### Synthesis of results

Results are presented as a narrative synthesis of data on the efficacy and components/characteristics of successful interventions to enhance cardiometabolic and psychosocial outcomes among PWH in sSA [2]. Where possible, the effects of the interventions on cardiometabolic and psychosocial health outcomes were assessed using the differences in pre- and post-test means. When not reported, we calculated these differences by subtracting the pre-test mean from the post-test mean. We also synthesised our results using the reported post-test results and the conclusions stated in the publication. In addition, the characteristics of the studies, including (a) name of author and year of publication (b) study design (c) intervention setting (d) outcomes assessed (e) characteristics of participants (f) description of the PA interventions and their efficacy are also presented in tables.

## Results

### Study selection

In total, 6 406 abstracts were retrieved by the search strategy (Fig 1). Nine hundred and ten duplicates were removed, and we were left with 5496 unique studies for abstract screening, of which 5478 were found to be irrelevant to the main subject of our study, i.e., 4,320 studies did not assess the efficacy of PA interventions in improving cardiometabolic or psychosocial outcomes but

**Fig 1:**
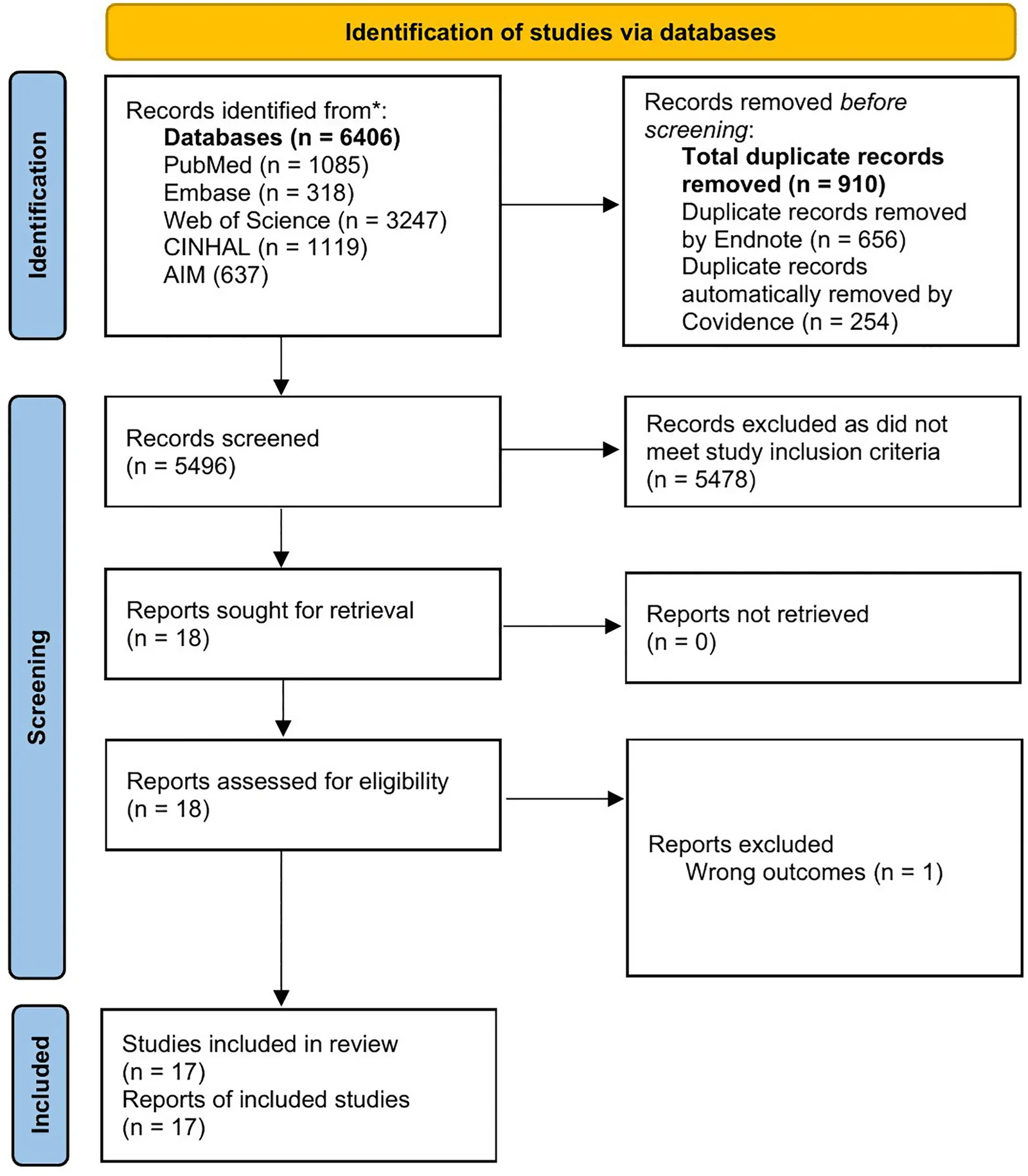
Flow diagram of the study selection process.

instead focused on other interventions, while 1,158 studies did not address our outcomes of interest. Eighteen studies were then assessed for eligibility through full manuscript review. One study [44] was excluded because the study did not assess any of our pre-specified outcomes of interest, leaving 17 studies that were included in the final systematic review.

### Study characteristics

Table 1 reports the characteristics of the studies included in this systematic review. Sixteen RCTs, and 1 quasi-experimental study were included. The included studies were conducted in Nigeria (n = 8), South Africa (n = 4), Zimbabwe (n = 2), Rwanda (n = 2), and Mozambique (n = 1). Studies were generally small, with sample sizes ranging from 21 to 100. Two studies focused on PWH with concurrent comorbidities, including one with HIV-related poly-neuropathy [45] and another on people with antiretroviral therapy-associated body fat redistribution syndrome [46]. Six of the 17 studies had cardiometabolic outcomes alone [45,47–51], eight had psychosocial outcomes alone [46,52–58] and three included both cardiometabolic outcomes and at least one psychosocial outcome [59–61]. In addition, two studies had PA as a behavioural outcome [54,60]. Cardiometabolic outcomes included blood pressure, body weight, waist circumference, hip circumference, waist-hip ratio, body fat, visceral fat, body mass index, skinfolds, cholesterol, LDLs, HDLs, triglycerides, blood glucose, HOMA-IR, and insulin. Psychosocial outcomes included depression, anxiety, perceived stress, body self-image, quality of life and self-efficacy. In the interventions that also targeted PA as a behavioural outcome, PA was assessed using pedometer step count in both studies [54,60], and in addition, Mabweazara [54] also assessed PA using the Global Physical Activity Questionnaire (GPAQ).

**Table 1:** Characteristics of the studies included in the systematic review.

Author Year, Country, Design	Intervention setting	Outcomes		Participants				
		Cardiometabolic	Psychosocial	n*	Age (mean, SD)	Female: n(%)**	ART Use	CD4 Count in cells/mm^3^ (mean, SD)
Aweto 2016, Nigeria, RCT	**---**	None	Depression	33	IG = 31 (6) CG = 32 (5)	23 (70%)	**✓**	NR
Daniels 2018, SA, RCT	Primary health care setting (Clinic)	None	Depression; Body self-image	60	39	60 (100%)^+^	**✓**	NR
Ezema 2014, Nigeria, RCT	University Physiotherapy Department	BP	None	33	IG = 39 (10) CG = 40 (10)	NR	**✓**	492 (230)mm^3#^
Ijego 2021, Nigeria, RCT	Primary health care setting (teaching hospital)	Body weight	None	38	NR	NR	**✓**	IG = 414 (198) CG = 461 (237)
John 2018, Nigeria, RCT	**---**	WC; BF; Visceral fat; BMI; BP	Quality of life	60	IG = 41 (8) CG = 41 (8)	33 (55%)	**✓**	NR
Mabweazara 2018, SA, RCT(crossover)	Community centre	None	Self-efficacy for PA	21	38 (9)	21 (100%)^+^	NR	470 (158)
Maduagwu 2017, Nigeria, RCT	Primary health care setting (teaching hospital)	None	Quality of life	64	IG = 41 (10) CG = 39 (10)	41 (64%)	**✓**	IG = 493 (145) CG = 484 (136)
Maharaj 2011, SA, RCT	Primary health care setting (hospital) and Home exercises	None	Quality of life	52	IG(female)=37 (9) IG(male)= 32 (11) CG(female)=29 (7) CG(male)=35 (10)	18 (35%)	**✓**	200-500cells/µl, *n (%)* IG(female): 6 (23) IG(male): 9 (35) CG(female): 3 (12) CG(male): 10 (38) ≥500cells/µl, *n (%)* IG(female): 4 (15) IG(male): 7 (27) CG(female): 5 (19) CG(male): 8 (31)
Mangona 2015, Mozambique, RCT	Exercise center	Body weight; BMI; WC; SFT; BF; BP; TC; HDL; TG; BG	None	53	40 (8)	53 (100%)^+^	**✓**	FEG: 525 (187) PEG: 445 (182) CG: 459 (206)
Mbayo 2020, Zimbabwe, RCT	NR	BMI; WHR; BF; FBG; FTC; BP	None	128	18-45	101 (79%)	**✓**	NR
Mkandla 2016, Zimbabwe, RCT	NR	None	Quality of life	160	42 (9)	NR	**✓59**%	*≥*200 cells/mm^3^, n (%) IG: 40 (50) CG: 45 (56) 201-400 cells/mm, n (%)^3^ IG = 12 (15) CG = 15 (19) *≥*401 cells/mm^3^, n (%) IG = 11(14) CG = 11(14)
Mutimura 2008, Rwanda, RCT	Fitness club	WC;WHR;SSKF;BF;HC;FTC;TG;BG; HDL;LDL;FBG; HOMA-IR; Insulin	None	97	IG = 38 (6) CG = 38 (7)	60 (62%)	**✓**	IG = 353 (168) CG = 348 (162)
Mutimura 2008, Rwanda, RCT	Fitness club	BFR score, BMI, WC,HC, WHR, SSKF, BF	Quality of life	100	IG = 38 (6) CG = 38 (7)	60 (60%)	**✓**	IG = 353 (168) CG = 348 (162)
Odunaiya 2002, Nigeria, Quasi experimental	Primary health care setting (clinic)	None	Depression, Anxiety, Self-efficacy	53	Age 20–29 (n = 2) Age 30–39 (n = 24) Age 40–49 (n = 27)	31 (59%)	**✓**	NR
Roos 2014, SA, RCT	Home-based pedometer walking program	WHR; BG;HDL;BMI;WC; HC; Body weight, C-reactive protein	Perceived stress	84	IG = 39 (9) CG = 39 (10)	66 (79%)	**✓**	IG: 340 (132) CG: 347 (172)
Shittu 2017, Nigeria, RCT	NR	None	Quality of life	30	IG: 37 (9) CG: 34 (7)	10 (33%)	**✓**	NR
Maduagwu 2015, Nigeria, RCT	NR	TG, HDL, LDL, TC	None	64	IG: 39 (10) C: 40 (10)	41 (64%)	**✓**	IG = 493 (145) CG = 484 (136)

*Participants that were randomised; **Females who completed the study; NR = Not reported; IG = intervention group; CG = Control group; SA = South Africa; + All participants were female; ^#^ mean(SD); FEG = Formal Exercise Group; PEG = Play Exercise Group; WC = Waist Circumference; SSKF = Sum skinfold thickness; BF = Body fat; BP = Blood pressure; HC = Hip Circumference; FTC = Fasting Total Cholesterol; TC = Total Cholesterol; FBG = Fasting Blood Glucose; WHR = Waist Hip Ratio; TG = Triglycerides; BG = Blood Glucose; HDL = High Density Lipoproteins; LDL = Low Density Lipoproteins; HOMA-IR = Homeostasis Model Assessment – Insulin Resistance; The data presented in this table were extracted by SZM and independently verified by MJS and JM-G. All studies included met the predefined eligibility criteria for inclusion in the review.

### Description of the interventions

Full description of the interventions and their efficacy on improving cardiometabolic and psychosocial health outcomes are presented in Table 2.

**Table 2:** Description of intervention components and their efficacy to improve cardiometabolic and psychosocial outcomes.

Study	Intervention Description	Control Group Intervention	Post-test Results	Conclusion and Effect
Aweto 2016	**Intervention:** Progressive aerobic exercise with warm up and cool down on a bicycle ergometer and counselling sessions. Gradual exercise loads administered incrementally based on 50–60% heart rate (HR) reserve (moderate intensity). **Frequency and Duration**: 30 min, 3 days a week for 6 weeks. The counselling sessions were for 30 min, once in two weeks. **Exercise modality**: Aerobic exercise	Group counselling sessions for 30 minutes every two weeks.	Significant improvement in depression in the IG while that of the CG showed no significant improvement.	Aerobic exercise training significantly reduced depressive symptoms in PWH.
Daniels 2018	**Intervention**: Structured, low impact exercise factoring in timing, technique, and degree of intensity. Participants taught how to warm up and cool down and how to avoid overexertion. Participants then took exercise booklets home to conduct prescribed exercises. Participants logged their exercise output in a logbook and recorded their weight, waist and hip circumference at the end of each week. **Frequency and Duration**: 6-week exercise program, twice a week. **Exercise modality**: NR	Informational brochure about PA	A relatively slight decrease in mean scores for depression in the IG and CG, as a result of the intervention. For the body self-image affective-attitudinal subscale, there was statistical significance in two subscales in the IG for negative affect and health fitness evaluation. The item of the cognitive-perceptual subscales which showed statistical significance was social desirability.	Depression was impacted positively by the therapeutic exercise program, as were certain aspects of body self-image.
Ezema 2014	**Intervention**: Progressive moderate intensity exercise of between 60% and 79% of HR reserve achieved by jogging on a treadmill. The exercise session was increased from 45 min in the first 2 weeks of training and levelled up at 60 min throughout the remaining part of the training. **Frequency and Duration**: 3 times/week for 8 weeks. **Exercise modality**: Aerobic exercise	None.	Significant decrease in SBP and DBP in the IG compared to the CG.	Moderate intensity aerobic exercise is an effective complementary therapy in lowering BP in PWH.
Ijego 2021	**Intervention**: Progressive resistance exercise that used 10 repetitive maximum of 10 percent of their body weight in progression. Exercise weights were progressively increased during the 6 weeks of the exercise with consideration of bone adaptation and fracture prevention. HR and blood pressure were monitored before and during each exercise bout. **Frequency and Duration**: 3 times a week for six weeks. **Exercise modality**: Resistance exercise	None.	Positive effect on weight which was not statistically significant.	The major findings of this study have revealed that progressive resistance exercise had a positive effect on weight.
John 2018	**Intervention**: Aerobic exercise intervention with nutritional guidance. Training performed progressively from an initial HR of 40% to 75% at a moderate intensity on a treadmill. Participants were instructed to rate their perceived exertion using the Borg Perceived Rate of Exertion Scale. Measurement of BP and HR were taken every 3 min as an additional monitor. **Frequency and Duration**: 3 times per week for maximum of 45 min for 6 weeks. **Exercise modality**: Aerobic exercise	Nutritional counselling	A significant difference in BMI, BF, BP VF, WC, respectively in both groups. In the IG there was a significant difference in all the body composition parameters except VF. All domains of QoL had a significant improvement in both groups	A 6-week aerobic exercise intervention coupled with nutritional guidance in PWH was able to significantly improve body composition and QoL.
Mabweazara 2018	**Intervention**: The intervention employed low-cost activities of daily living (ADLs) using local resources and opportunities. The PA intervention comprised a combination of PA educational activities (60 min), home-based activities (30 min) and a 30-minute group aerobics session. The IG received logbooks for recording daily PA, and a PA manual with information on home-based exercise activities, the benefits and barriers to PA, and strategies for improving exercise self-efficacy. Food packs were used to promote adherence. PA was assessed using the GPAQ and total weekly steps using pedometers. **Frequency and Duration**: 2 hours/wk for 6 weeks. **Exercise modality**: Multimodal exercise	Nutritional information on healthy eating in a single 2-hour session with an informational brochure.	Significant increases in exercise self-efficacy in the IG.	The intervention was effective in producing significant increases in PA in a sample of PWH of low SES over six weeks.
Maduagwu 2017	**Intervention**: Progressive moderate intensity treadmill aerobic exercise, from 50% to 75% of HR at 12 weeks for 20 minutes. The exercise comprised of a warm up phase with stretching exercises, and finally, a cool down phase. The training exercise protocol was supervised by two physical therapists, an exercise physiologist, and a physician. **Frequency and Duration:** 12 weeks of moderate intensity exercise, three times per week for 40 minutes per session. **Exercise modality**: Aerobic exercise	Documented lectures once a week on issues pertaining to nutrition, meaning of HIV and AIDS, relationship and differences between HIV and AIDS, mode of HIV transmission and its prevention, and adherence to ARV therapy.	The QoL domains of the IG were significantly higher than the values before the beginning of the study. In the CG, the result shows that pre-and post-tests mean scores of physical functioning, role limitation due to physical health, role limitation due to emotional health and bodily pain of the participants did not differ significantly. In contrast, the mean scores of energy/ fatigue, emotional wellbeing, social functioning, and general health were significantly lower at the end of the study.	The quality of life of PWH in the IG improved significantly after 12 weeks of moderate intensity aerobic exercise.
Maharaj 2011	**Intervention**: The IG cycled on an ergometer set with minimal resistance for two sessions of 10 min of cycling and 5 min of rest. This was followed by two sessions of 10 min of treadmill walking with 5 min of rest. Both of these were set at 50–70% of their HR. The home protocol was 10 min each of brisk walking, squatting with the hands on the hips, and jogging on the spot three times per week. Participants were taught how to manually monitor their respiratory rates, HRs, and BP using a digital BP monitor. The total duration of exercises per session was 40 minutes with 20 min of rest. **Frequency and Duration:** once a week over 3 months, resulting in 12 exercise sessions. The home protocol was 10 min of exercise three times per week. **Exercise modality**: Multimodal exercise	Pulsed shortwave diathermy to quadriceps and hamstring muscles bilaterally for 10 min each with minimal intensity, which served as a placebo, followed by 10 minutes of rest. The home program for the CG was reading a magazine for 30 minutes three times per week.	All QoL domains improved significantly for the IG compared with the CG. Both the physical component scores and the mental component scores improved significantly from the baseline following the rehabilitation program.	These results indicate that a rehabilitation program of moderate-intensity exercises with a home program significantly improved the QoL for individuals on HAART.
Mangona 2015	**Intervention**: The study included two groups: the formal exercise group (FEG) with 19 participants and the playful exercise group (PEG) with 18 participants. Both groups underwent a 13-week exercise program. The FEG engaged in aerobic and circuit weight training, progressively increasing intensity. The circuit comprised six exercises with 15 repetitions each. The PEG participated in a 15-minute warm-up, a 40-minute main session involving diverse aerobic group activities like dancing, skipping, and pre-sports games, and a 5-minute cool-down. The PEG activities emphasized energy, coordination, socialization, and psychological skills. **Frequency and Duration:** 3 times/week of one hour duration. **Exercise modality**: Multimodal exercise	None.	Mean weight and BMI increased in all groups and was statistically significant for CG. WC reduced significantly in PEG while skinfolds reduced only in in CG. With the exception of cholesterol, which increased in the IGs, no differences were founded in BP, cholesterol, HDL, TG, and BG.	It was concluded that there is no contraindication for exercise in this type of population and the beneficial effect was mainly in cardiorespiratory fitness, regardless of the type of exercise performed.
Mbayo 2020	**Intervention**: Participants in the (IG) engaged in supervised resistance exercise sessions targeting major muscle groups: biceps, triceps, chest, calf, hamstrings, and quadriceps. The program followed a periodization approach, gradually increasing intensity from 50% to 80% of each individual’s 1RM by week 12. Sessions were supervised by sport scientists, commencing with warm-ups and concluding with cool-down activities involving dynamic and static stretches. Monetary support was provided to ensure session attendance. **Frequency and Duration:** Three days per week for 12 weeks. **Exercise modality**: Resistance exercise	None.	Significant improvements in resting BP and body composition (lean body mass, percentage body fat, fat mass, waist-to-hip ratio), and significantly reduced FBG, FTC, and resting BP in the IG compared to the CG. Furthermore, a noteworthy 66% of IG participants demonstrated enhanced resting blood pressure values, indicating positive progress from baseline to week 12.	The intervention programme had a significant effect on PWH receiving cART. Improvements in body composition and chronic disease risk values were noted in the IG compared to the CG at the end of the intervention.
Mkandla 2016	**Intervention**: Participants in the IG underwent a lower limb progressive resistance exercise (PRE) intervention (quadriceps, hamstring, tibialis anterior and gastrocnemius). The exercise programme consisted of warm up, stretching and PRE. **Frequency and Duration**: Twice weekly for 12 weeks. **Exercise modality**: Resistance exercise	Health promotion advice about engaging in physical activity.	**QoL**: The exercise intervention significantly improved health-related QoL in the IG when compared to the CG measured using the state of health visual analogue, but not when using the utility score.	Progressive resistance exercises have some positive effects on the HR-QOL in subjects with HIV/ AIDS-related Distal symmetrical poly-neuropathy.
Mutimura 2008a	Participants in the IG participated in a supervised exercise training program at a Fitness Club. All participants were instructed on a progressive exercise program consisting of stretching exercises and 15 min of brisk walking followed by 45–60 min of jogging, running, stair climbing, low back and abdominal stabilization, and strengthening exercises. Participants were instructed to perform primarily jogging and running exercises with a goal of achieving at least 45% age-predicted maximal heart rate (HR) in the first 3 weeks, 60% age-predicted maximal HR in the next 6 weeks, and 75% age-predicted maximal HR by the end of the intervention. Each exercise training session ended with 15 min of cool down and stretching exercises. **Frequency and Duration**: 6-months, (3 sessions/week, 1.5 h/session, alternating days) **Exercise modality**: Multimodal exercise	None.	BMI, WC, and WHR declined more in the IG, while it remained unchanged or increased in the other groups. HC was unchanged in all groups. Subscapular, suprailiac, and sum skinfold thicknesses declined more in the IG than in all other groups. There was a significant decrease in % BFM. TC levels declined more in the IG than the HIV ^+^ BFR ^+^ noEXS group. There were no significant changes in HDL, LDL, and HOMA values, but insulin levels declined more in the IG than in the HIV ^+^ BFR ^+^ noEXS group. Significant decrease in fasting plasma glucose in IG than HIV ^+^ BFR ^+^ noEXS. Interestingly, HIV ^+^ BFR ^+^ noEXS had a significant increase in fasting plasma glucose, whereas HIV^-^noEXS subjects did not. Also, the serum TG level declined more in the IG than in HIV ^+^ BFR ^+^ noEXS and was not changed in other subject groups	Exercise training positively modulated body composition and metabolic profiles in HAART-treated HIV Africans.
Mutimura 2008b	**Intervention**: Participants in the intervention group engaged in a 6-month supervised PA program (three sessions/week, 1.5 h/session) at a fitness club in Kigali. The program included warmup, stretching, 15 min of brisk walking, and 45–60 min of jogging, running, stair climbing, low-back, and abdominal stabilization and strengthening exercises. The exercise regimen was progressive, aiming for at least 45% age-predicted maximum heart rate in the first 3 weeks, 60% in the next 6 weeks, and 75% by the end of the intervention. Daily heart rate assessment guided exercise intensity, ensuring cardiorespiratory and musculoskeletal adaptations. Sessions concluded with a 15-min cool down and stretching. Adherence was monitored, with nonadherence defined as 50% missed sessions. **Frequency and Duration**: 6-months, (3 sessions/week, 1.5 h/session, alternating days) **Exercise modality**: Multimodal exercise	None.	**QoL**: significant improvement in QoL measures in the IG compared with BFR + noEXS subjects. In particular, significant improvements were observed in the IG for the psychological, independence, social relationships, and HIV+ HAART-specific domains of QoL. Specific improvements in psychological well-being were observed for self-esteem and satisfaction with social life, body image and appearance and emotional stress. Although the overall QoL domain improved in the IG, we observed larger relative improvements in the psychological, independence, social relations, and HIV HAART-specific domains of life. There was no significant difference in the changes in scores on the physical domain of QoL between the IG and BFR + noEXS groups.	Exercise training improved several components of QoL in HAART-treated HIV+ African subjects with BFR.
Odunaiya 2002	**Intervention**: Participants in the IG underwent an aerobic exercise training programme alongside HAART. The programme, which was supervised by a physiotherapist, started with a five-minute warm-up session and ended with a five-minute cooldown session. The workout comprised brisk walking, jogging on a spot, jumping jack exercise and race within a defined area. **Frequency and Duration**: Participants in the experimental group carried out exercise for thirty minutes, three times weekly for four weeks. **Exercise modality**: Aerobic exercise	None.	At the end of the intervention, there was significant improvement in depression, anxiety and self-efficacy scores in the IG compared to the control group.	More attention should be given to the prevention and management of psychological problems in PWH through the use of aerobic exercise.
Roos 2014	**Intervention**: Participants in the IG underwent an educational and home-based pedometer progressive walking program. They were provided with a pedometer and an activity diary containing educational material and self-monitoring tools. Five monthly clinic sessions were conducted to discuss diary content, covering topics such as ischemic heart disease, associated risk factors in HIV + , benefits of physical activity (PA), intensity monitoring during walking, and community safety. Personalized step count goals were set, with regular adjustments and SMS motivation. The incremental walking program began with a 1000-step/day increase thrice weekly, progressing to 3000 steps. Stretching exercises, pulse palpation, and breathlessness monitoring were taught. Brisk walking at 60%-75% heart rate and moderate Borg Scale rating was encouraged. Intervention adherence and pedometer usage were assessed at study completion. **Frequency and Duration**: 12 months with 5 monthly contact sessions. **Exercise modality**: Aerobic exercise	Perceived stress	**WHR; BG; HDLs**: Significant between-group effects observed on WHR *(P = 0.00)*, BG *(P = 0.00)*, and HDLs *(P = 0.01)*.	An education and home-based pedometer walking program improves PA levels, and beneficial changes in other IHD risk factors were noted.
Shittu 2020	**Intervention**: The IG received aerobic exercise training in addition to taking their regular medication. Aerobic exercise was performed on a bicycle ergometer. Participants exercised at an intensity of 50–60% of maximum heart rate (MHR), which represents a moderate intensity aerobic exercise. Each exercise training session lasted for about 30 minutes to about 45 minutes. Participants who became exhausted, breathless, had feelings of discomfort, or felt that they could no longer continue were asked to discontinue training. **Frequency and Duration**: Participants in the IG exercised at a frequency of at least 3 times/week for about 30 minutes to about 45 minutes and for a duration of 6 weeks. **Exercise modality**: Aerobic exercise	None.	**QoL**: The scores of PCS and MCS sub-scales of QoL of the IG significantly increased compared to the CG.	Aerobic exercise is potentially beneficial in improving functional capacity and health status of PLWHA.
Maduagwu 2015	Intervention; The IG participated in progressive moderate intensity treadmill aerobic exercise, with 5 minutes of warm up phase, 30 minutes conditioning/training phase and 5 minutes of cool down phase) per session. The exercise intensity was between 50 and 75% of heart rate reserve (HRR). The target training heart rate (TTHR)/training intensity was pegged at 50% of HRR from 1st week– 2nd week and increased to 75% at the 11th - 12th week. **Frequency and Duration**:12 weeks, three times per week for 40 minutes. **Exercise modality**: Aerobic exercise	Organized lectures once a week on issues pertaining to nutrition, meaning of HIV and AIDS, relationship and differences between HIV and AIDS, mode of HIV transmission and its prevention, and adherence to ARV therapy.	There was a significant improvement in Chol, TG, HDL and LDL between pre- and post-tests in the IG compared to the CG.	The lipid profile of HIV infected persons who participated in the 12 weeks moderate intensity treadmill aerobic exercise significantly improved.

*Only based on the outcomes of interest; IG: Intervention group; CG: Control group; HR: Heart rate; BF: Body fat; BP: Blood Pressure; VF: Visceral fat; ARV: Antiretroviral therapy; HAART: Highly Active Antiretroviral therapy; QoL: Quality of life; RM: Repetition Maximum; BMI: Body mass index; BFM: Body fat mass; WC: Waist circumference; HC: Hip circumference; WHR: Waist Hip Ratio; TC: Total cholesterol, FBG: Fasting Blood Glucose; FTC: Fasting total cholesterol; HDL: High density lipoproteins; LDL: Low density lipoproteins; TG: Triglycerides; BG: Blood glucose; FBG: Fasting blood glucose; SBP: Systolic blood pressure; DBP: Diastolic blood pressure; Ins: Insulin; HOMA_IR_: homeostasis model assessment of insulin resistance; NR:Not reported; BFR: Body fat redistribution; PCS: Physical Component Score; MCS: Mental Component Score; The data presented in this table were extracted by SZM and independently verified by MJS and JM-G. All studies included met the predefined eligibility criteria for inclusion in the review.

### Physical activity intervention trials with cardiometabolic health outcomes

Most (7/8) of the interventions included structured interventions with supervised training plans, whereas one was self-led [60]. The trials with cardiometabolic outcomes evaluated PA interventions with resistance exercise (n = 2) [48,50], aerobic exercise (n = 4) [47,51,59,60] or a combination of aerobic and resistance exercise (n = 2) [45,49]. Most interventions set goals for moderate exercise intensity (i.e., 40–79% of maximum heart rate) with gradual increases in intensity as the intervention progressed. The interventions set exercise goal ranges from 45-90 minutes per session, with sessions occurring 3 times/week (7/8), and total intervention durations of 6–12 weeks (n = 6), and 6 months (n = 2). Neither the locations of exercise nor the supervisor was reported in most (5/8) studies. Two studies evaluated gym-based programs [45,50] and another focused on a home-based exercise strategy [60]. Finally, only one study included education on information on cardiometabolic health risk factors [60] in the intervention and no study included diet or lifestyle modification interventions in addition to PA.

### Physical activity intervention trials with psychosocial health outcomes

Most (7/11) of the interventions included structured interventions with supervised training plans. The trials with psychosocial outcomes evaluated PA interventions with resistance exercise (n = 1) [46], aerobic exercise (n = 6) [52,55,57–60] or a combination of aerobic and resistance exercise (n = 3) [54,56,61].One study did not report the exercise mode used in the intervention [53]. Most interventions set goals for moderate exercise intensity (i.e., 50–75% of maximum heart rate) with gradual increases in intensity as the intervention progressed. The interventions set exercise goal ranges from 30-90 minutes per session, with sessions occurring 3 times/week (8/11), and total intervention durations in the 6–12 week range(6/11). Five studies did not report exercise location, but 3 studies had a home exercise component [53,54,60]; one study [54] also had a community-centre based component and the last [61] evaluated a gym-based program. Exercise supervision was not reported in 4 studies, but the intervention was self-led in 2 studies [53,60], a component led by an exercise scientist in one study [54], and led by physiotherapists in two studies [57,61]. Finally, 3 studies included educational information on cardiometabolic health [60] and PA [53,54]. Only one study incorporated incentivisation as a strategy to promote and maintain participant adherence [54]. This involved providing fruit packs and food parcels to participants allocated to the exercise intervention arm, which were distributed contingent upon attendance at each scheduled contact session during the intervention period. Exercise monitoring was reported in 3 studies using log books [53,54,60], and pedometers [54,60].

### Physical activity intervention trials with physical activity participation outcomes

Two interventions [54,60] had PA outcomes in addition to cardiometabolic and psychosocial outcomes and are also listed in Table 2. The intervention in one [54], was delivered in the indigenous Xhosa language and included a community centre-based program and a home-based exercise program. At the community centre, participants were taught simple home-based exercise activities, strategies for increasing walking step count, increasing PA through activities of daily living, role of PA for NCD prevention in PWH, health benefits of PA for PWH, safety when exercising, PA self-efficacy and social support, identification and strategies for overcoming barriers to PA. Participants also engaged in a 30-minute group aerobic exercise session. Participants then received a PA manual with all the taught material and were then instructed to regularly do the home-based exercise activities and walk more frequently. Exercise monitoring was via a pedometer and a PA log book. In another study that measured PA [60], participants attended 5 monthly contact sessions where they were taught about ischaemic heart disease (IHD), risk factors for IHD, benefits of PA, strategies for increasing PA, monitoring PA intensity when walking, and safety suggestions when walking in the community. Participants then received a home-based walking program which was monitored via a pedometer and an activity diary.

### Effects of physical activity interventions on health outcomes

The numerical results of selected studies are presented in Table 3 for cardiometabolic outcomes and Table 4 for psychosocial outcomes, while Table 5 presents the efficacy of the PA interventions to improve cardiometabolic and psychosocial outcomes.

**Table 3:** Numeric results of selected studies with cardiometabolic outcomes.

Studies With Cardiometabolic Outcomes								
Study	Variable	Intervention Group			Control Group			P-Value*
		Pre-test Mean (SD)	Post-test Mean (SD)	*Δ Pre to Post*	Pre-test Mean (SD)	Post-test Mean (SD)	*Δ Pre to Post*	
**Ijego (2021)** ^**#**^	Weight (kg)	69.02 (12.58)	71.09 (13.04)	2. 07	67.08 (17.84)	67.57(17.15)	0.49	NR
**John (2018)** ^**#**^	SBP (mmHg)	123 (15.78)	77.47 (13.38)	-45.53	125 (15.67)	124.20 (14.54)	-0.8	0.001*
	DBP (mmHg)	77 (10.52)	71.3 (6.11)	-5.7	78.9 (10.49)	77.7 (9.79)	-1.2	0.001*
	BMI (kg/m^2^)	27.35 (5.10)	23.37 (3.55)	-3.98	27.35 (5.10)	27.2 (5.10)	-0.15	0.002*
	BF (%)	30.52 (10.56)	27 (10.00)	-3.52	30.92 (9.92)	30.6 (10.10)	-0,32	0.042*
	VF (%)	6.9 (1.61)	6 (1.37)	-0.9	7.93 (1.64)	7.59 (1.68)	-0.32	0.001*
	WC (cm)	82.53 (8.58)	77.1 (6.59)	-5.43	81.5 (8.58)	81.27 (8.49)	-0.23	0.002*
**Mangona (2015)** ^**#**^								
-Formal exercise group	Weight (kg)	63.4 (15.0)	64.9 (14.6)	1.5	59.9 (7. 1)	61.3 (7.5)	1.4	0.006*
	BMI (kg.m^-2^)	24.6 (5.2)	25.3 (4.9)	0.7	23.4 (2.9)	23.9 (3.1)	0.5	0.180
	WC (cm)	82.6 (13.4)	82.9 (13.1)	0.3	84.4 (83.0)	84.9 (8.3)	0.5	0.880
	SKF (cm)	134.0 (53.6)	123.1 (35.4)	-10.9	123.3 (35.8)	107.3 (30.0)	-16	0.140
	BF (%)	30.8 (9.1)	31.5 (8.6)	0.7	29.1 (7.1)	30.0 (7.4)	0.9	0.260
	SBP (mmHg)	125.0 (13.4)	124.7 (14.4)	-0.3	120.4 (17.2)	119.8 (17.3)	-0.6	0.440
	DBP (mmHg)	79.1 (8.6)	78.2 (8.7)	-0.9	78.4 (10.0)	78.47 (10)]	0.07	0.330
	Chol (mg/dL)	174.3 (40.9)	183.9 (43.5)	9.6	177.4 (34.1)	180.7 (30.7)	3.3	0.030*
	HDL (mg/dL)	52.3 (16.3)	54.2 (14.7)	1.9	48.7(11.3)	50.4 (18)	1.7	0.570
	TG (mg/dL)	84.2 (40.5)	93.5 (46.2)	9.3	91.1 (37.1)	106.0 (31.3)	14.9	0.230
	BG (mmol/L)	81.0 (23.3)	74.2 (17.3)	-6.8	74.2 (37.4)	78.3 (7.7)	4.1	0.310
-Play exercise group	Weight (kg)	61.6 (12.0)	62.3 (11.3)	0.7	59.9 (7.1)	61.3 (7.5)	1.4	0.200
	BMI (kg.m^-2^)	24.1 (4.8)	24.4 (4.5)	0.3	23.4 (2.9)	23.9 (3.1)	0.5	0.210
	WC (cm)	84.1 (12.6)	81.0 (10.6)	-3.1	84.4 (83.0)	84.9 (8.3)	0.5	0.008*
	SKF (cm)	126.4 (53.3)	116.0 (37.8)	-10.4	123.3 (35.8)	107.3 (30.0)	-16	0.170
	BF (%)	30.5 (8.2)	29.05 (9.7)	-1.45	29.1 (7.1)	30.0 (7.4)	0.9	0.070
	SBP (mmHg)	118.2 (15.1)	118.2 (15.2)	0	120.4 (17.2)	119.8 (17.3)	-0.6	0.660
	DBP (mmHg)	77.3 (11.3)	77.1 (11.3)	-0.2	78.4 (10.0)	78.47 (10)	0.07	0.330
	Chol (mg/dl)	150.9 (40.0)	174.6 (39.5)	23.7	177.4 (34.1)	180.7 (30.7)	3.3	0.004*
	HDL (mg/dl)	50.7 (19.1)	56.8 (19.6)	6.1	48.7 (11.3)	50.4 (18)	1.7	0.060
	TG (mg/dL)	81.7 (55.8)	88.4 (40.4)	6.7	91.1 (37.1)	106.0 (31.3)	14.9	0.410
	BG (mmol/L)	71.9 (16.8)	84.1 (13.7)	12.2	74.2 (37.4)	78.3 (7.7)	4.1	0.007*
**Mbayo (2020**)^#^	Weight (kg)	68.04 (15.94)	67.50 (15.21)	-0.54	65.97 (8.14)	66 (8.15)	0.03	<0.0005*
	BMI (kg/m^2^)	23.64 (5.74)	23.44 (5.48)	-0,54	24.63 (3.58)	24.63 (3.58)	0	<0.0005*
	BF (%)	21.27 (10.01)	19.82 (9.52)	-1.45	22.63 (6.64)	22.70 (6.3723)	0.07	<0.0005*
	FM (kg)	15.67 (10.21)	14.42 (9.53))	-1.25	15.11 (5.52)	14.67 (5.96)	-0.44	<0.0005*
	WC (cm)	84.34 (11.51)	82.20 (11.03)	-2.14	77.90 (13.59)	79.32 (13.52)	1.42	<0.0005*
	HC (cm)	99.00 (13.95)	98.54 (13.57)	-0.46	94.56 (16.11)	93.81 (16.39)	-0.75	0.051
	WHR	0.86 (0.09)	0.84 (0.08)	-0.02	0.83 (0.11)	0.84 (0.11)	0.01	<0.0005*
	FBG (mmol/L)	5.58 (1.95)	5.39 (1.18)	-0.19	5.57 (0.93)	5.86 (0.83)	0.29	<0.0005*
	FTBC (mmol/L)	4.44 (0.53)	4.24 (0.49)	-0.2	4.55 (0.45)	4.67 (0.50)	0.12	<0.0005*
	SBP (mmHg)	126.75 (21.33)	121.51 (21.39)	-5.24	108.2 7 (10.13)	110.77 (10.59)	2.5	<0.0005*
	DBP (mmHg)	84.53 (13.24)	82.92 (13.26)	-1.61	79.08 (6.41)	81.44 (6.93)	2.36	<0.0005*
**Mutimura (2008a)**	BMI (kg/m^2^)	24.0 (3.1)	NR	-0.53 (1.2)	24.4(2.7)	NR	0.06 (0.6)	<0.0001*
	WC (cm)	91.0 (8.4)	NR	-7.13 (4.4)	92.3(6.9)	NR	0.03 (1.7)	<0.0001*
	HC (cm)	92.3 (7.6)	NR	1.13 (5.4)	93.7(6.8)	NR	0.22 (0.7)	NR
	WHR	0.99 (0.1)	NR	-0.10 (0.1)	0.98(0.0)	NR	0.00 (0.1)	<0.0001*
	BFR score _(7 item)_	193 (2.1)	NR	-4.7 (0.9)	18.7 (2.3)	NR	0.51 (1.3)	<0.0001*
	SSKF (mm)	62.3 (15.0)	NR	-6.15 (8.2)	62.7 (12.9)	NR	-1.25 (3.5)	<0.0001*
	BFM (%)	29.4 (6.2)	NR	-1.5 (3.3)	29.3 (4.3)	NR	-0.16 (0.7)	<0.0001*
	TC (mmol/L	3.78 (0.93)	NR	-0.03 (1.11)	3.89 (1.05)	NR	0.0661 (1.28)	<0.95
	HDL (mmol/L)	1.28 (0.19)	NR	0.03 (0.21)	1.27 (0.15)	NR	0.07 (0.14)	NR
	LDL (mmol/L)	2.10 (0.74)	NR	0.14 (0.89)	2.16 (0.77)	NR	0.19 (0.980)	NR
	TG (mmol/L)	1.33 (0.39)	NR	-0.22 (0.48)	1.34 (0.36)	NR	0.07 (0.49)	<0.95
	BG (mmol/L)	4.76 (0.76)	NR	-0.21 (0.71)	4.95 (0.74)	NR	0.39 (1.25)	<0.0001*
	Ins (*p*mol/L)	67 (29)	NR	-1 (18)	4.95 (0.74)	NR	0.39 (1.25)	<0.95
	HOMA_IR_	2.04 (0.14)	NR	0.0 (0.1)	1.87 (0.13)	NR	0.0 (0.2)	NR
**Mutimura (2008b)**	BFR score _(7 item)_	19.3 (2.1)	NR	-4.7 (0.9)	18.7 (2.3)	NR	0.51 (1.3)	<0.0001*^
	BMI (kg/m^2^)	24.0 (0.1)	NR	-0.53 (0.2)	24.5 (0.42)	NR	0.1 (0.1)	<0.05*^
	WC (cm)	91.0 (8.4)	NR	-7.13 (4.4)	92.3 (6.9)	NR	0.03 (10.7)	<0.0001*^
	HC (cm)	92.3 (7.6)	NR	1.13 (5.4)	93.7 (6.8)	NR	0.22 (0.7)	0.19*^
	WHR	0.99 (0.1)	NR	-0.10 (0.1)	0.98 (0.0)	NR	0.00 (0.1)	<0.0001*^
	SSKF (mm)	62.3 (2.1)	NR	-6.15 (1.2)	61.2 (1.7)	NR	-1.25 (0.5)	<0.0001*^
	BFM (%)	29.4 (0.92)	NR	-1.5 (0.5)	29.3 (0.6)	NR	-0.16 (0.1)	<0.0001*^
**Roos (2014)**	SBP (mm. Hg)	121.29 (1.62)	NR	-2.52 (1.17)	120.11 (61.98)	NR	-1.71 (1.49)	0.14
	DBP (mm. Hg)	79.36 (1.25)	NR	-1.05 (1.07)	78.21 (1.34)	NR	--0.94 (1.15)	0.26
	Weight (kg)	70.10 (1.39)	NR	1.06 (0.75)	69.92(2.89)	NR	0.94 (0.58)	0.13
	BMI (kg/m^2^)	25.88 (0.62)	NR	1.56 (0.35)	25.36 (1.00)	NR	1.39 (2.51)	0.33
	WC (cm)	81.26 (1.14)	NR	0.38 (0.71)	79.83 (1.94)	NR	0.03 (0.51)	0.09
	HC (cm)	104.02 (1.52)	NR	0.65 (0.78)	103.51 (2.46)	NR	-0.39 (0.49)	0.00*
	WHR	0.78 (0.02)	NR	-0.01 (0.01)	0.77 (0.01)	NR	0.003 (0.004)	0.00*
	BG (mmol/L)	4.81 (0.11)	NR	-0.11 (0.2)	5.10 (0.27)	NR	-0.18 (0.10)	0.00*
	TC (mmol/L)	4.31 (0.13)	NR	-0.02 (0.09)	4.35 (0.16)	NR	-0.21 (0.08)	0.11
	HDL (mmol/L)	1.42 (0.08)	NR	0.09 (0.05)	1.41 (0.06)	NR	0.01 (0.03)	0.01
	LDL (mmol/L)	2.45 (0.10)	NR	-0.13 (0.07)	2.53 (0.13)	NR	-0.24 (0.07)	0.57
	TG (mmol/L)	0.98 (0.08)	NR	0.02 (0.06)	1.02 (0.07)	NR	0.03 (0.05)	0.50
**Maduagwu (2015)** ^**#**^	TC (mg/dL)	214.56 (52.49)	156.23 (35.58)	-58.33	222.84 (47.51)	224.29 (52.98)	1.45	<0.001*
	TG (mg/dL)	180.36 (93.23)	113.41 (60.25)	-66.95	198.47 (62.21)	207.32 (80.63)	8.85	<0.001*
	HDL (mg/dL)	35.86 (12.04)	57.67 (47.95)	21.81	31.13 (10.27)	29.78 (9.67)	-1.35	<0.001*
	LDL (mg/dL)	128.07(65.71)	73.09 (25.91)	-54.98	134.16 (42.76)	135.73 (39.44)	1.57	<0.001*

NB: *Significance level set at *P* < 0.05; *^Significance level set at *P* value ≤0.05; Δ Pre to Post: Differences in pre and post-intervention means; BMI: Body mass index; BFM: Body fat mass; WC: Waist circumference; HC: Hip circumference; WHR: Waist Hip Ratio; TC: Total cholesterol, FTC: Fasting total cholesterol; HDL: High density lipoproteins; LDL: Low density lipoproteins; TG: Triglycerides; BG: Blood glucose; FBG: Fasting blood glucose; SBP: Systolic blood pressure; DBP: Diastolic blood pressure; Ins: Insulin; HOMA_IR_: homeostasis model assessment of insulin resistance; NR:Not reported; ^#^:indicates studies were differences in pre and post-intervention means were not present in the published articles and were calculated by subtracting the pre-test mean from the post-test mean; The data presented in this table were extracted by SZM and independently verified by MJS and JM-G. All studies included met the predefined eligibility criteria for inclusion in the review.

**Table 4:** Results of selected studies with psychosocial outcomes.

STUDIES WITH PSYCHOSOCIAL OUTCOMES						
STUDY	VARIABLE	INTERVENTION GROUP		CONTROL GROUP		P-value*
		Pre-test Mean (SD)	Post-test Mean (SD)	Pre-test Mean (SD)	Post-test Mean (SD)	
**Aweto(2016)**#	Depression	10.33(6.48)	3.50(1.27)	10.06(5.96)	833(5.80)	0.001*
**Daniels(2018)**#	Attention to grooming	12.23(1.45)	11.78(1.79)	11.939(2.04)	11.34(3.59)	NR
	investment in ideals	11.63(1.77)	11.10 (1.56)	11.60(1.39)	10.27(3.21)	NR
	health/fitness influence	12.16(1.68)	12.50 (1.29)	11.83(1.57)	11.20 (3.49)	NR
	Negative Affect	7.46(2.37)	6.46 (2.08)	6.43(1.90)	5.62 (2.49)	NR
	Health/fitness evaluation	9.36(1.60)	10.53 (1.59)	10.63(1.56)	9.44 (3.08)	NR
	Overall appearance evaluation	11.00 (2.00)	11.89 (1.85)	12.30 (1.62)	11.79 (2.09)	NR
	Height dissatisfaction	3.66 (0.92)	4.46 (1.45)	4.10 (1.09)	3.75 (1.66)	NR
	Social desirability	9.20 (1.09)	8.00 (1.82)	8.46 (1.61)	7.44 (2.75)	NR
	Fatness evaluation	6.90 (2.30)	5.92 (1.60)	6.10 (1.42)	5.17 (2.08)	NR
	Depression	6.17(3.97)	4.85 (2.85)	4.64 (2.79)	2.75 (2.10)	NR
**John(2018)**	General health	26.17(5.68)	51.33(3.93)	21.17(6.11)	35(5.41)	0.001*
	Physical functioning	38.97(4.97)	92.47(6.45)	40.1(5.33)	67.1(9.00)	0.001*
	Role functioning	20(24.90)	73.33(31.44)	25(25.43)	41.67(32.37)	0.001*
	Cognitive functioning	42.7(17.90)	100(0.00)	34.83(9.10)	72.67(8.17)	0.001*
	Mental health	23.77(11.33)	71.37(3.71)	27.33(11.43)	60.37(6.67)	0.001*
	Energy/vitality	26.67(5.46)	59.67(4.72)	29.5(8.13)	47(7.38)	0.001*
**Odunaiya(2002)**#	Depression	20.52(5.75)	13.92(4.79)	23.28 (4.64)	25.21(5.89)	< 0.001*
	Anxiety	11.56(3.40)	7.04(2.44)	13.28 (3.72)	12.92(3.38)	< 0.001*
	Self-efficacy	25.28(4.79)	26.60 (6.45)	23.57(6.13)	23.00(6.03)	0.04*
**Mutimura(2008b)**			***Δ at Post***		***Δ at Post***	
	Physical	16.9 (0.7)	0.2 (0.1)	17.2 (0.4)	0.0 (0.1)	0.57
	Psychological	11.1 (0.5)	1.3 (0.3)	11.0 (0.3)	0.5 (0.1)	<0.0001*^
	Independence	17.5 (0.6)	0.6 (0.1)	17.6 (0.7)	0.0 (0.0)	<0.0001*^
	Social relationships	9.2 (0.5)	0.6 (0.2)	9.0 (0.5)	0.0 (0.0)	<0.0001*^
	HIV HAART-specific	4.7 (0.4)	1.4 (0.2)	4.7 (0.6)	-0.1 (0.2)	<0.0001*^
	Overall QoL	15.6 (0.7)	0.5 (0.3)	15.5 (1.1)	0.0 (0.3)	<0.0001*^
**Shittu(2020)**#	Physical Component Score	41.3(5.88)	48.7(5.06)	41.3(7.49)	41.5(7.32)	0.004*
	Mental Component Score	47.5(8.83)	55.6(6.23)	46.9(7.91)	47.6(8.72)	0.007*
**Roos(2014)**			***Δ at Post***		***Δ at Post***	
	Perceived stress	20.12 (61.03)	-1.76(1.02)	20.14 (60.99)	-0.98 (1)	0.61*

NB: NR: Not reported; FEG: formal exercise group; PEG: play exercise group; *Significance level set at *P* < 0.05; *^Significance level set at *P* value ≤0.05; ^#^: indicates studies were differences in pre and post-intervention means were not present in the published articles and were calculated by subtracting the pre-test mean from the post-test mean. The data presented in this table were extracted by SZM and independently verified by MJS and JM-G. All studies included met the predefined eligibility criteria for inclusion in the review.

**Table 5:** Efficacy of the physical activity interventions to improve cardiometabolic and psychosocial outcomes.

	Outcomes																						
	Cardiometabolic																Psychosocial						Behavioural
Study	BP	BW	WC	HC	WHR	BF	VF	BMI	SF	CHL	LDL	HDL	TG	BG	HOM	INS	DP	ANX	PS	BSI	QoL	SE	PA
Aweto 2016																							
Daniels 2018																				#			
Ezema 2014																							
Ijego 2021																							
John 2018																							
Mabweazara 2018																							
Maduagwu 2017																							
Maharaj 2011																							
Mangona 2015			##																				
Mbayo 2020																							
Mkandla 2016																					**		
Mutimura 2008a																							
Mutimura 2008b																							
Odunaiya 2002																							
Roos 2014																							
Shittu 2020																							
Maduagwu 2015																							

Green: Positive improvement of outcome in intervention group at the end of the intervention

Orange: Non-significant improvement of outcome in intervention group at the end of the intervention

Red: No or negative impact on outcome in intervention group at the end of the intervention

BP = blood pressure; BW = Body weight; HC = Hip circumference; WHR = waist hip ratio; BF = body fat; VF = visceral fat; BMI = body mass index; SF = skinfolds; CHL = cholesterol; LDL = low density lipoproteins; HDL = high density lipoproteins; TG = triglycerides; BG = blood glucose; HOM = ; INS = insulin; DP = depression; ANX = Anxiety; PS = perceived stress; BSI = body self-image; QoL = Quality of Life; SE = self-image. *: Means impacted positively by PA but not statistically significant; #: Means only certain aspects of the outcome were impacted; ##: Means only in the play exercise group of the intervention group; **: Means only when measured using the state of health visual analogue, but not when using the utility score; The data presented in this table were extracted by SZM and independently verified by MJS and JM-G. All studies included met the predefined eligibility criteria for inclusion in the review.

### Effects of physical activity interventions on cardiometabolic health outcomes

The most commonly evaluated cardiometabolic health indicators were blood pressure (n = 5) [47,49,50,59,60], body weight (n = 3) [48,49,60], BMI (n = 4) [45,49,59,60], body and visceral fat (n = 5) [45,49,50,59,61], waist circumference (n = 4) [45,49,59–61], cholesterol (n = 4) [45,49–51], HDLs (n = 4) [45,49,51,60], triglycerides (n = 3) [45,49,51], glucose (n = 4) [45,49,50,60], and waist-hip-ratio (n = 4) [45,50,60,61]. By and large, PA interventions evaluated in these studies had positive impacts on cardiometabolic health indicators (**Tables 2 and 3**). For example, all 8 studies that evaluated the efficacy of PA interventions to improve cardiometabolic health outcomes reported at least one positive impact. The most common improvements reported were on reducing blood glucose in 3 of 4 studies [45,50,60], waist circumference in 3 of 4 studies [45,49,59], body fat in 3 of 3 studies [32,45,50], cholesterol in 3 of 4 studies [45,50,51], waist hip ratio in 3 of 3 studies [45,50,60], triglycerides in 2 of 3 studies [45,51], and blood pressure in 2 of 3 studies [47,50]. Two studies reported no impact on some cardiometabolic outcomes: one [49] reported no impact of a formal exercise intervention and a playful exercise intervention on blood pressure, HDLs, triglycerides, and blood glucose, whilst another [45] reported that a cardiorespiratory training exercise intervention had no impact on hip-circumference, HDLs, LDLs, and HOMA-IR. Using an education and home-based pedometer walking program, one study [60] reported a negative impact on weight, waist circumference, and BMI, whilst another study [49] also reported negative impacts on weight, BMI, and cholesterol.

### Effects of physical activity interventions on psychosocial health outcomes

The most commonly evaluated psychosocial health indicators were quality of life (n = 6) [46,55,56,58,59,61], depression (n = 2) [53,57], and self-efficacy (n = 2) [54,57]. While a single study evaluated perceived stress [60], and another evaluated body self-image [53]. All 11 studies that focused on psychosocial health reported a positive impact on the outcomes of interest (Tables 2 and 4). However, in one study [46], the positive impact of the intervention was restricted to quality of life as measured using the state of health visual analogue scale, and only certain aspects of body self-image in the one study which incorporated that measure [53].

### Effects of physical activity interventions on physical activity outcomes

Table 2 also lists the studies that evaluated the effects of PA interventions on PA outcomes. Only two studies assessed PA outcomes [54,60]. A positive impact on PA was reported for pedometer step count in both studies. Additionally, one of the studies [54] reported a positive impact on PA (metabolic equivalent/week) as measured by the GPAQ.

### Behavioural change techniques identified in physical activity interventions promoting physical activity

S1 Table shows a detailed description of the BCTS identified in the studies that had an effect on PA outcomes. The following BCTs were identified in one study [54]: information about health consequences, feedback on the behaviour, feedback on outcomes of the behaviour, goal-setting outcome, self-monitoring of the behaviour, material incentive (behaviour), demonstration of the behaviour, problem solving, instruction on how to perform the behaviour, social support (unspecified), social support (practical), goal-setting behaviour, review the behavioural goals, and action planning. Whereas in the other study evaluating PA promotion [60]: information about health consequences, feedback on the behaviour, goal-setting outcome, self-monitoring of the behaviour, problem solving, and instruction on how to perform the behaviour were all reported.

### Methodological quality

The included RCTs generally scored high on the PEDro scale, ranging from 7 [53] to 10 [46,56,60]. Risks identified included absence of blinding of participants, therapists, and assessors who measured key outcomes. Only 3 studies reported blinding of therapists and assessors [46,56,60]. Baseline comparability was challenging in three studies [48,49,53]. Randomisation, concealed allocation, adequate follow-up, intention-to-treat analysis, between-group comparisons, and reporting point estimates and variability were present in all studies. In summary, PEDro scores were as follows: 10/11, n = 3 [46,56,60]; 8/11, n = 9 [45,47,50,52,54,55,58,59,61]; 7/11, n = 2 [48,49]; and 6/11, n = 1 [53] (Table 6). The study by Odunaiya [57] was a non-randomized quasi-experimental study, assessed using the Downs and Black checklist, and scored 12 out of 20, which was considered a good quality score (S2 Table). The study scored well in reporting on items 1–10 (7/10), did not score any points on external validity on items 11–13 (0/3), and scored all relevant items (items 14–20) for internal validity.

**Table 6:** Quality assessment of the studies using the PEDro scale.

	Eligibility criteria	Random allocation	Concealed allocation	Baseline comparability	Blind subjects	Blind therapists	Blind assessors	Adequate follow-up	Intention to- treat analysis	Between-group comparisons	Point estimates And variability	Total Score
Aweto 2016	1	1	1	1	0	0	0	1	1	1	1	8/11
Daniels 2018	0	1	1	0	0	0	0	1	1	1	1	6/11
Ezema 2014	1	1	1	1	0	0	0	1	1	1	1	8/11
Ijego 2021	1	1	1	0	0	0	0	1	1	1	1	7/11
John 2018	1	1	1	1	0	0	0	1	1	1	1	8/11
Mabweazara 2018	1	1	1	1	0	0	0	1	1	1	1	8/11
Maduagwu 2017	1	1	1	1	0	0	0	1	1	1	1	8/11
Maharaj 2011	1	1	1	1	0	1	1	1	1	1	1	10/11
Mangona 2015	1	1	1	0	0	0	0	1	1	1	1	7/11
Mbayo 2020	1	1	1	1	0	0	0	1	1	1	1	8/11
Mkandla 2016	1	1	1	1	0	1	1	1	1	1	1	10/11
Mutimura 2008a	1	1	1	1	0	0	0	1	1	1	1	8/11
Mutimura 2008b	1	1	1	1	0	0	0	1	1	1	1	8/11
Roos 2014	1	1	1	1	0	1	1	1	1	1	1	10/11
Shittu 2020	1	1	1	1	0	0	0	1	1	1	1	8/11
Maduagwu 2015	1	1	1	1	0	0	0	1	1	1	1	8/11

NB: Odunaiya [41] not included because the study was a quasi-experimental study and thus, methodological quality of the study was conducted using the Downs and Black checklist reported in S2 Table.

## Discussion

We retrieved 16 randomized controlled trials and one quasi-experimental trial to examine effects of PA on cardiometabolic and psychosocial health among PWH. Improvements in cardiometabolic health outcomes were reported across a range of markers, including blood pressure, lipids, BMI, body weight, waist circumference, and body fat. Likewise, improvements in psychosocial outcomes included augmented quality of life, reduction in depression, improved self-efficacy, decreased perceived stress, and improved body self-image. Notably, the largely positive impact of PA interventions on health was seen despite the heterogeneity of the PA interventions evaluated, which ranged from structured and supervised programs to individual and community-based models, and from relatively modest interventions, such as 30-to-40-minutes of aerobic workouts 3 times/week for six weeks, to much more substantial interventions, such as 1.5 hours of combined aerobic and resistance training, 3 times/week for 6-months. A key limitation of most trials was the relatively short duration (e.g., 6–12 weeks), making it impossible to detect major clinical events. Notwithstanding, this body of work offers strong preliminary support for the use of PA interventions to improve cardiometabolic and psychosocial health among PWH, and argues for additional research to evaluate these interventions at larger scale.

Physical activity interventions have been studied for their effectiveness in improving cardiometabolic and psychosocial outcomes among PWH. A systematic review by O’Brien et al. [16] analysed 24 randomized controlled trials (RCTs) involving 936 participants, predominantly male and on antiretroviral therapy. The interventions, which included aerobic exercise alone or combined with resistance training, ranged from 5 to 52 weeks in duration. The findings indicated significant improvements in cardiorespiratory fitness, strength, body composition, and quality of life among participants who engaged in these PA interventions compared to non-exercisers. Notably, no significant changes were observed in CD4 counts or viral load, suggesting that PA interventions are safe and beneficial for medically stable adults living with HIV. A systematic review and meta-analysis by Kalatzi et al. [62] examined the effects of supervised aerobic exercise on clinical, physiological, and mental health parameters in PWH. Findings revealed that supervised aerobic exercise significantly reduced depressive symptoms. However, the meta-analysis found no significant changes in CD4 T-cell count or blood pressure, indicating that aerobic exercise did not have a substantial impact on these immunological and hemodynamic markers.

Another systematic review and meta-analysis by Aminde et al. [63] evaluated the effectiveness of minimally supervised home and community exercise (HCE) interventions in PWH. The analysis encompassed 13 studies involving 857 participants aged between 29 and 56 years. The interventions primarily consisted of aerobic and strength exercises performed with minimal supervision in home or community settings. The results showed that HCE interventions led to significant improvements in PA levels, with an increase of approximately 1,097 steps per day compared to control groups. Additionally, there was a notable reduction in body fat percentage by 3.36%, although no significant change was observed in body mass index (BMI). Quality of life (QoL) also improved, particularly in the physical health domain, with an increase of 13 points relative to controls. Notably, interventions conducted at moderate or higher intensities were associated with greater benefits, and walking-only programs were found to be at least as effective as other forms of exercise. Importantly, no adverse events were reported across the included studies.

Lastly, an umbrella review [64] prepared for the Guideline Development Group for the WHO Guidelines on PA and sedentary behaviour for children and adolescents, adults and older adults highlights the significant advantages of regular PA for PWH. Results revealed that engaging in appropriately graded exercise can help preserve lean body mass, enhance cardiopulmonary function, bolster immune status, and improve overall quality of life. Additionally, PA was shown to improve quality of life, cardiometabolic health, and cardiorespiratory fitness in this population. Although not specifically focused for PWH in sub-Saharan Africa, the evidence for the benefits of PA shown in these systematic reviews shows that incorporating regular PA is crucial for optimising the health and well-being of PWH.

In our study, a greater proportion of participants were female. Similarly, a review examining the health benefits of PA for Black women, including African Black women [65], reported that PA improved cardiometabolic and psychosocial outcomes, as well as benefits for pregnancy- and menopause-related conditions. African women encounter distinct challenges and bear a higher risk of obesity compared to men and women in the Global North usually shaped by a complex interplay of individual, cultural, social, economic, and environmental factors [66]. In South Africa, research shows that 60% of African women are either overweight or obese, with obesity rates among women being five times higher than those observed in men [67]. Based on our findings, PA appears to be an effective therapeutic approach for improving health outcomes, although the considerable heterogeneity among studies warrants cautious interpretation.

In our study, the included studies discussed in the discussion section scored well on the PEDro scale (≥9/11 points). However, most fell short in blinding subjects, therapists, and assessors [45,50,54,55,58–61], as well as in external validity [57]. Perhaps the most compelling feature of this body of evidence is the consistency in improvements seen across multiple CMD and psychosocial endpoints as shown in Table 5. Essentially every study showed a benefit of PA, and the benefits were wide ranging although of relatively modest effect size. All studies examining blood pressure demonstrated potentially significant improvements. The study by John et al. [59] reported the most substantial improvements, with reductions in systolic blood pressure by 46 mmHg and diastolic blood pressure by 6 mmHg. Notably, reviews of randomized controlled trials of PA interventions have also demonstrated relatively modest changes in blood pressure (4 and 2 mm Hgb, respectively) [68]. Thus, these data are consistent with data outside the HIV literature. Similarly, although one study by Maduagwu et al [55] showed large improvements in lipids (i.e., a 22 mg/dl increase in HDL and a 55mg/dl decrease in LDL), others, such as that by Roos et al [41], Mbayo [50], and Mutimura [45] were associated with much smaller changes. Smaller differences were also observed in a prior systematic review with nine randomized controlled trials which, although not focusing in PWH in sSA, found that various exercise types had modest but meaningful benefits in terms of lipid profiles and blood glucose levels in PWH [69]. Finally, improvements in body composition outcomes were also observed, ranging from large reductions in BMI (i.e., a decrease of 4 kg/m^2^) to a 4% reduction in body fat in the study by John et al. [59], > 5% body weight reduction in Mutimura et al. [45,61], a 7.13 cm reduction in waist circumference in both studies by Mutimura [45,61], and 0.2 kg/m^2^ reduction in BMI, 0.5 kg reduction in body weight, 2 cm reduction in waist-circumference, and 1% reduction in body fat in the study by Mbayo [50]. These magnitudes of change are consistent with general population reviews that demonstrate that regular exercise significantly improves body composition parameters [70–75]. The impact of PA on body composition are particularly important in the era of modern ART, which has been associated with significant increases in weight in PWH [9].

It is important to note that some studies reported null effects on certain cardiometabolic outcomes despite improvements in others. For example, Mutimura et al. [45] found improvements in central adiposity, cardiorespiratory fitness, triglycerides, and fasting glucose, but no significant changes in HDL-C, LDL-C, or HOMA-IR following the PA intervention. Across methodological scrutiny and comparison with adjacent evidence, these null findings are likely explained by several factors, including: limited baseline risk (“room to improve”), as HDL and LDL levels were not markedly abnormal; study design and power limitations, with analyses not powered for HDL, LDL, or HOMA-IR and reliance on per-protocol rather than intention-to-treat approaches; measurement constraints, including the use of calculated LDL using the Friedewald equation, which is known to possess significant limitations in terms of accuracy [76] and the limited sensitivity of HOMA-IR [77]; and biological influences of HIV and ART, which may attenuate improvements in lipid profiles and insulin resistance [78]. Importantly, similar trials often report no change in HDL, suggesting this finding is not unusual. Improvements in LDL and insulin resistance appear more likely when baseline abnormalities are greater, exercise dose and intensity are tightly controlled, or more sensitive measures of insulin sensitivity are used [79].

Mangona et al. [49] reported “no exercise effect” on somatic (weight/BMI/body fat/most anthropometrics) and most cardiovascular risk variables (blood pressure, HDL, triglycerides) improvements in cardiorespiratory fitness (VO₂_peak_). The null findings may be best explained by a convergence of (i) limited baseline impairment and ceiling effects (BP, glucose, lipids, and CD4 were generally in relatively normal/stable ranges), (ii) an intervention that likely prioritized fitness over energy deficit/body-composition change (only ~20 min structured aerobic work per session in the formal group; resistance emphasized endurance with low volume), (iii) statistical design/analysis choices (small per-group number of participants, no reported power calculation or primary outcome hierarchy; reliance on within-group paired tests rather than a formal (interaction model) testing whether changes over time differ between groups (e.g., intervention vs control).

Importantly, the benefit of PA interventions extended beyond CMD outcomes. Moreover, the benefits were consistent across all studies that considered psychosocial outcomes, even if the outcomes differed. For example, in Odunaiya et al [57], an aerobic exercise programme led to improvements in depression, anxiety, and self-efficacy. Although the outcomes differed, the studies by Mutimura et al [61] and Shittu et al [58] reported improvements in quality of life. These data are consistent with prior reviews in this area. For example, another systematic review [16] not focused on PWH in sSA on the efficacy of aerobic exercise for PWH reported statistically significant improvements in selected outcomes of depression and quality of life in the intervention groups than controls. These results are consistent with an extensive meta-review (97 reviews, 1039 trials, and 128,119 participants) in the general population, which found PA to be highly beneficial for improving symptoms of depression, anxiety, and distress across various adult populations, including those with mental health disorders and chronic diseases [80]. In that review, some of the greatest benefits in terms of psychosocial outcomes were seen among PWH. Interestingly, in that review briefer exercise programs provided greater benefit than extended regimens, and the benefits diminished with longer-duration programs. In our review, interventions to improve mental health conditions and quality of life also had relatively short durations ranging from 30 minutes in Odunaiya et al [57] and Shittu et al [58] to 1.5 hours in Mutimura et al [61]. On balance, these data suggest that a maximum therapeutic benefit of PA on mental health conditions may be drawn from shorter duration PA activities.

Only two studies in our review incorporated interventions to specifically promote PA among PWH [54,60]. Both interventions were effective in promoting PA, measured as step-count captured via pedometers [54,60] and as self-reported in the Global Physical Activity Questionnaire [54]. Emphasizing increased step-count through walking [54,60] and incorporating self-led home exercise activities and activities of daily living [54], these interventions align with the positive outcomes of a meta-analysis showing the efficacy of walking interventions, especially in group settings, for increasing PA in adults [81]. Both interventions evaluated in our review implemented activity diaries for self-monitoring and included an educational component aimed at increasing understanding of the health benefits of PA for PWH. Common BCTs incorporated in the interventions included providing information about health consequences, feedback on behaviour, goal setting, self-monitoring, problem solving, and instruction on how to perform the behaviour

(S1 Table). Notably, these interventions had an unsupervised home-based component, an educational component, and were not overly rigorous, which may enhance their potential for feasibility and future scalability.

A prominent limitation in many PA intervention studies is the lack of robust long-term follow-up and limited evidence of sustained behavioural change beyond the active intervention period. Although acute increases in PA are commonly achieved during intervention delivery, maintaining these gains and translating them into sustained health benefits remains challenging. Beyond identifying effective BCTs during intervention delivery, greater attention is needed on the role of maintenance strategies to support long-term effects and sustainability. Evidence from long-term reviews suggests that interventions incorporating tailored exercise prescriptions and systematic follow-up, such as booster sessions (e.g., phone calls, text messages, in-person follow-up sessions, or tailored reminders), ongoing contact (e.g., follow-up prompts and self-monitoring), behavioural counselling, or tailored self-management support, are more effective in reinforcing PA behaviour beyond the intervention period and demonstrate better maintenance of PA behaviour compared with interventions that do not include such strategies [82,83].

### Limitations of the current review

The studies evaluated were of modest quality. Nonetheless, the interventions in our review varied widely in terms of target populations, types of interventions, delivery personnel, measurement tools, and duration, which prevented the ability to consider pooling the data. Limitations of the study included short follow-up periods, absence of measurements for maintenance after the initial intervention period, absence of active controls, and lack of blinding of study staff when assessing outcomes. This lack of blinding is particularly concerning for interventions targeting psychosocial outcomes. Additionally, small sample sizes and observation period prevented estimation of intervention effects on clinical outcomes such as cardiovascular events. Such data may ultimately be needed to help ensure PA interventions are ultimately adopted at scale and in public health guidelines. We also acknowledge that while the PEDro scale can be helpful in identifying potential methodological issues, it cannot definitively confirm the presence of publication bias without further analysis considering factors such as the overall publication landscape.

### Future research implications

Future research should focus on well-powered, longitudinal studies to determine whether the short-term improvements in cardiometabolic and psychosocial outcomes observed among PWH in sSA are sustained over time and translate into reductions in hard clinical endpoints such as cardiovascular disease, diabetes, and HIV-related multimorbidity. Larger, multi-site trials across diverse and resource-limited settings are needed to enhance generalisability, alongside the use of objective PA measures to improve assessment accuracy. Further studies should identify the key intervention components and BCTs responsible for observed benefits and apply implementation science approaches to evaluate feasibility, scalability, cost-effectiveness, and integration of PA interventions into routine HIV care, while also addressing equity-related factors such as sex, age, treatment regimen, comorbidities, and socio-environmental determinants of PA participation.

### Policy and practice implications

The consistent evidence of cardiometabolic and psychosocial benefits highlights the need to integrate structured PA promotion into HIV care policies and clinical guidelines in sSA, recognising PA as a core component of comprehensive HIV management alongside ART. Health systems should support task-shifting approaches that enable trained lay health workers and community-based providers to deliver low-cost, culturally appropriate PA interventions within existing HIV programmes, strengthening holistic and person-centred care. At the policy level, sustained investment and cross-sectoral collaboration are essential to support scalable, community-based PA initiatives and create supportive environments, particularly in underserved settings, with the potential to improve long-term health outcomes and quality of life among PWH.

## Conclusions

In conclusion, amidst rising concerns about CMD consequences of lifelong HIV infection in the current ART era, this review supports further study of PA to improve CMD risk and psychosocial health among PWH. While preliminary, our results suggest that PA interventions could be a valuable addition to standard HIV care packages in sSA, offering an alternative approach to enhance both cardiometabolic and psychosocial well-being in this population, particularly at high risk for obesity and metabolic conditions [84]. Future research should explore the most effective exercise modalities, intervention durations and intensities to elicit positive improvements in both cardiometabolic and psychosocial aspects among PWH. Importantly, focus should be on the use of effective BCTs to develop feasible, context-sensitive interventions that can be scaled up to promote PA among PWH, especially in low socioeconomic settings or amongst underserved populations.

## Supporting information

S1 Appendix Search strategy. (DOCX)

S1 Checklist PRISMA checklist. (DOCX)

S1 Table Behavioural change techniques used in effective interventions with an intention to promote physical activity among people living with HIV. (DOCX)

S2 Table Quality assessment of the study by Odunaiya et al. (2022) using the Downs and Black checklist. (DOCX)

S3 Table Articles after-full text review and reasons for exclusion. (DOCX)
