# Supplementary material for: Physical activity interventions for cardiometabolic and psychosocial outcomes in people living with HIV in sub Saharan Africa: A systematic review
Source: PLOS Glob Public Health. 2026 Sep 28;6(9):e0007070. doi: 10.1371/journal.pgph.0007070 (PMC13618956; doi:10.1371/journal.pgph.0007070)
Supplement: Supplementary file 1 [file pgph.0007070.s001.docx]

**SEARCH STRATEGY**

| Last search run: August 10^th^, 2023  Title: Effects of physical activity interventions on cardiometabolic and psychosocial outcomes in people living with HIV in sub-Saharan Africa: A systematic review | | |
| --- | --- | --- |
| Database | Search Strategy | Result |
| PubMed | (((("Exercise"[Mesh] OR "Sports"[Mesh] OR "Physical Fitness"[Mesh] OR "Cardiorespiratory Fitness"[Mesh] OR "Movement"[Mesh] OR interval[tiab] OR lifestyle*[tiab] OR physical activit*[tiab] OR PA intervention*[tiab] OR endurance[tiab] OR weight-lifting[tiab] weight-bearing[tiab] OR high-intensity[tiab] OR HIIT[tiab] OR cardiorespiratory[tiab] OR cardio[tiab] OR resistance[tiab] OR strength[tiab] OR athletic*[tiab] OR working-out[tiab] OR work-out[tiab] OR strength-training[tiab] OR sport*[tiab] OR team[tiab] OR steps[tiab] or pedometer[tiab] OR aerobic*[tiab] OR muscle-strengthening[tiab] OR bone-strengthening[tiab] OR stretching[tiab] OR exercise*[tiab]) AND (Active[tiab] OR activit*[tiab] OR fitness[tiab] OR training[tiab] OR exercis*[tiab] OR lifting[tiab] OR conditioning[tiab] OR intervention*[tiab] OR physical[tiab] OR physically[tiab] OR exercise*[tiab] OR physical activit*[tiab])) AND ((Human[tiab] AND Immunodeficiency[tiab] AND Virus[tiab]) OR (Acquired[tiab] AND Immune[tiab] AND Deficiency[tiab] AND Syndrome[tiab]) OR “Human Immunodeficiency Virus” OR “Acquired Immune Deficiency Syndrome Virus” OR AIDS[tiab] OR AIDS Virus[tiab] OR Seronegativities[tiab] OR Seronegativity[tiab] OR HIV[tiab] OR hiv-1[tiab] OR hiv-2[tiab] OR hiv1[tiab] OR hiv2[tiab] OR hiv infect*[tiab] OR “human immune deficiency virus”[tiab] OR “human immuno-deficiency virus”[tiab] OR “human immune-deficiency virus”[tiab] OR “acquired immuno-deficiency syndrome”[tiab] OR “acquired immune-deficiency syndrome”[tiab] OR HIV/AIDS[tiab] OR HIV-positive[tiab] OR HIV[ti] OR HIV-AIDS[ti] OR "HIV"[Mesh] OR "HIV Long-Term Survivors"[Mesh] OR "HIV Seronegativity"[Mesh] OR "HIV Infections"[Mesh] OR "HIV Seroprevalence"[Mesh] OR "HIV-2"[Mesh] OR "HIV-1"[Mesh] OR "HIV Seropositivity"[Mesh] OR HIV[tiab])) AND ("Africa"[Mesh] OR "Africa South of the Sahara"[Mesh] OR Africa*[tiab] OR Subsaharan[tiab] OR sub-saharan[tiab] OR Cameroon[tiab] OR Central African Republic[tiab] OR Chad[tiab] OR Congo[tiab] OR Democratic Republic Congo[tiab] OR Equatorial Guinea[tiab] OR Gabon[tiab] OR “Sao Tome and Principe” OR Burundi[tiab] OR Djibouti[tiab] OR Eritrea[tiab] OR Ethiopia[tiab] OR Kenya[tiab] OR Rwanda[tiab] OR Somalia[tiab] OR South Sudan[tiab] OR Sudan[tiab] OR Tanzania[tiab] OR Uganda[tiab] OR Angola[tiab] OR Botswana[tiab] OR Eswatini[tiab] OR Lesotho[tiab] OR Malawi[tiab] OR Mozambique[tiab] OR Namibia[tiab] OR South Africa[tiab] OR Zambia[tiab] OR Zimbabwe[tiab] OR Benin[tiab] OR Burkina Faso[tiab] OR Cabo Verde[tiab] OR “Cote d'Ivoire” OR Gambia[tiab] OR Ghana[tiab] OR Guinea[tiab] OR Guinea-Bissau[tiab] OR Liberia[tiab] OR Mali[tiab] OR Mauritania[tiab] OR Niger[tiab] OR Nigeria[tiab] OR Senegal[tiab] OR Sierra Leone[tiab] OR Togo[tiab] OR Xhosa[tiab] OR Xosa[tiab] OR Bantu[tiab] OR Rundi[tiab] OR Rwanda[tiab] OR Shona[tiab] OR Zulu[tiab] OR Lingala[tiab] OR Swahili[tiab] OR Hausa[tiab] OR Yoruba[tiab] OR Ibo[tiab] OR Igbo[tiab] OR Kongo[tiab] OR Fulani[tiab] OR Amharic[tiab] OR Oromo[tiab] OR Chewa[tiab] OR Sotho[tiab] OR Showa[tiab] OR Khoisan[tiab] OR KhoiSan[tiab] OR Khoi-San[tiab] OR Khoe-San[tiab] OR Malagasy[tiab] OR Afrikaans[tiab] OR Hausa[tiab] OR Songhay[tiab] OR Songhai[tiab] OR Berber[tiab])) AND ("clinical trial"[pt] OR "random allocation"[Mesh] OR random*[tiab] OR clinical trial[tiab] OR clinical study[tiab] OR controlled trial[tiab] OR controlled study[tiab] OR "Pilot Projects"[Mesh] OR ((phase i[tiab] OR phase 1[tiab] OR phase 2[tiab] OR phase 3[tiab]) AND (study[tiab] OR studies[tiab] OR trial[tiab])) OR program*[tiab] OR intervention[tiab] OR pilot[tiab] OR strateg*[tiab]) | 1085 |
| Embase | No. \| Query \| Results; #9 \| #7 AND #8 \| **318**; #8 \| 'randomized controlled trial'/exp OR 'clinical study'/exp OR 'pilot study'/exp OR 'types of study'/exp OR 'clinical trial':ab,ti OR random*:ab,ti OR 'clinical trial*':ab,ti OR 'clinical study':ab,ti OR 'controlled trial*':ab,ti OR 'controlled study':ab,ti OR (('phase i*':ab,ti OR 'phase 1*':ab,ti OR 'phase 2*':ab,ti OR 'phase 3*':ab,ti OR trial:ab,ti OR trials:ab,ti) AND (study:ab,ti OR studies:ab,ti OR trial:ab,ti)) OR program*:ab,ti OR intervention*:ab,ti OR pilot*:ab,ti OR strateg*:ab,ti \| **32866630**; #7 \| #3 AND #6 \| **320**; #6 \| #4 AND #5 \| **10843**; #5 \| ('africa'/exp OR 'africa south of the sahara'/exp OR africa*:ab,ti OR africa*:ti OR subsaharan:ab,ti OR 'sub saharan':ab,ti OR cameroon:ab,ti OR 'central african republic':ab,ti OR chad:ab,ti OR congo:ab,ti OR 'democratic republic of congo':ab,ti OR 'equatorial guinea':ab,ti OR gabon:ab,ti OR 'sao tome and principe' OR burundi:ab,ti OR djibouti:ab,ti OR eritrea:ab,ti OR ethiopia:ab,ti OR kenya:ab,ti OR rwanda:ab,ti OR somalia:ab,ti OR 'south sudan':ab,ti OR sudan:ab,ti OR tanzania:ab,ti OR uganda:ab,ti OR angola:ab,ti OR botswana:ab,ti OR eswatini:ab,ti OR lesotho:ab,ti OR malawi:ab,ti OR mozambique:ab,ti OR namibia:ab,ti OR 'south africa':ab,ti OR zambia:ab,ti OR zimbabwe:ab,ti OR benin:ab,ti OR burkina) AND faso:ab,ti OR 'cape verde':ab,ti OR 'cote d ivoire' OR gambia:ab,ti OR ghana:ab,ti OR guinea:ab,ti OR 'guinea bissau':ab,ti OR liberia:ab,ti OR mali:ab,ti OR mauritania:ab,ti OR niger:ab,ti OR nigeria:ab,ti OR senegal:ab,ti OR 'sierra leone':ab,ti OR togo:ab,ti OR xhosa:ab,ti OR xosa:ab,ti OR bantu:ab,ti OR rundi:ab,ti OR rwanda:ab,ti OR shona:ab,ti OR zulu:ab,ti OR lingala:ab,ti OR swahili:ab,ti OR yoruba:ab,ti OR ibo:ab,ti OR igbo:ab,ti OR kongo:ab,ti OR fulani:ab,ti OR amharic:ab,ti OR oromo:ab,ti OR chewa:ab,ti OR sotho:ab,ti OR showa:ab,ti OR khoisan:ab,ti OR 'khoi san':ab,ti OR 'khoe san':ab,ti OR malagasy:ab,ti OR afrikaans:ab,ti OR hausa:ab,ti OR songhay:ab,ti OR songhai:ab,ti OR berber:ab,ti \| **255166**; #4 \| (('human immunodeficiency virus' OR (human:ab,ti AND immunodeficiency:ab,ti AND virus:ab,ti) OR (acquired:ab,ti AND immune:ab,ti AND deficiency:ab,ti AND syndrome:ab,ti) OR 'acquired immune deficiency syndrome virus' OR aids:ab,ti OR aids) AND virus:ab,ti OR seronegativities:ab,ti OR seronegativity:ab,ti OR hiv:ab,ti OR 'hiv 1':ab,ti OR 'hiv 2':ab,ti OR hiv1:ab,ti OR hiv2:ab,ti OR hiv) AND infect*:ab,ti OR 'human immune deficiency virus':ab,ti OR 'human immuno-deficiency virus':ab,ti OR 'human immune-deficiency virus':ab,ti OR 'acquired immuno-deficiency syndrome':ab,ti OR 'acquired immune-deficiency syndrome':ab,ti OR 'hivaids':ab,ti OR 'hiv positive':ab,ti OR hiv:ti OR 'hiv aids':ti OR 'human immunodeficiency virus'/exp OR 'hiv survivor'/exp OR 'hiv test'/exp OR 'human immunodeficiency virus infection'/exp OR 'human immunodeficiency virus prevalence'/exp OR hiv:ab,ti \| **580692**; #3 \| #1 AND #2 \| **1082977**; #2 \| active:ab,ti OR activit*:ab,ti OR fitness:ab,ti OR training:ab,ti OR exercis*:ab,ti OR lifting:ab,ti OR conditioning:ab,ti OR intervention*:ab,ti OR physical:ab,ti OR physically:ab,ti OR exercise*:ab,ti OR 'physical activit*':ab,ti \| **7842787**; #1 \| ('exercise'/exp OR 'physical activity, capacity and performance'/exp OR 'sports and sport related phenomena'/exp OR 'sport'/exp OR 'fitness'/exp OR 'cardiorespiratory fitness'/exp OR 'movement (physiology)'/exp OR interval:ab,ti OR lifestyle*:ab,ti OR 'physical activit*':ab,ti OR 'pa intervention*':ab,ti OR endurance:ab,ti OR 'weight lifting':ab,ti) AND 'weight bearing':ab,ti OR 'high intensity':ab,ti OR hiit:ab,ti OR cardiorespiratory:ab,ti OR cardio:ab,ti OR resistance:ab,ti OR strength:ab,ti OR athlet*:ab,ti OR 'working out':ab,ti OR 'work out':ab,ti OR 'strength training':ab,ti OR sport*:ab,ti OR team:ab,ti OR steps:ab,ti OR pedometer:ab,ti OR aerobic*:ab,ti OR 'muscle strengthening':ab,ti OR 'bone strengthening':ab,ti OR stretching:ab,ti OR exercise*:ab,ti \| **2532778** | 318 |
| Web of Science  Core Collection  Editions = A&HCI, BKCI-SSH, BKCI-S, CCR-EXPANDED, ESCI, IC, CPCI-SSH, CPCI-S, SCI-EXPANDED, SSCI | **1**  **AB=(("Exercise" OR "Sports" OR "Physical Fitness" OR "Cardiorespiratory Fitness" OR "Movement" OR interval OR lifestyle* OR “physical activit*” OR “PA intervention*” OR endurance OR weight-lifting weight-bearing OR high-intensity OR HIIT OR cardiorespiratory OR cardio OR resistance OR strength OR athletic* OR working-out OR work-out OR strength-training OR sport* OR team OR steps or pedometer OR aerobic* OR muscle-strengthening OR bone-strengthening OR stretching OR exercise*) AND (Active OR activit* OR fitness OR training OR exercis* OR lifting OR conditioning OR intervention* OR physical OR physically OR exercise* OR “physical activit*”) )**  **2,115,972**  2  AB= ((Human AND Immunodeficiency AND Virus) OR (Acquired AND Immune AND Deficiency AND Syndrome) OR “Human Immunodeficiency Virus” OR “Acquired Immune Deficiency Syndrome Virus” OR AIDS OR AIDS Virus OR Seronegativities OR Seronegativity OR HIV OR hiv-1 OR hiv-2 OR hiv1 OR hiv2 OR "hiv infect*" OR “human immune deficiency virus” OR “human immuno-deficiency virus” OR “human immune-deficiency virus” OR “acquired immuno-deficiency syndrome” OR “acquired immune-deficiency syndrome” OR HIVAIDS OR HIV-positive OR HIV OR HIV-AIDS OR "HIV" OR "HIV Long-Term Survivors" OR "HIV Seronegativity" OR "HIV Infections" OR "HIV Seroprevalence" OR "HIV-2" OR "HIV-1" OR "HIV Seropositivity" OR HIV)  673,379  3  AB= ("Africa" OR "Africa South of the Sahara" OR Africa* OR Subsaharan OR sub-saharan OR Cameroon OR Central African Republic OR Chad OR Congo OR Democratic Republic of the Congo OR Equatorial Guinea OR Gabon OR “Sao Tome and Principe” OR Burundi OR Djibouti OR Eritrea OR Ethiopia OR Kenya OR Rwanda OR Somalia OR South Sudan OR Sudan OR Tanzania OR Uganda OR Angola OR Botswana OR Eswatini OR Lesotho OR Malawi OR Mozambique OR Namibia OR South Africa OR Zambia OR Zimbabwe OR Benin OR Burkina Faso OR Cabo Verde OR “Cote d'Ivoire” OR Gambia OR Ghana OR Guinea OR Guinea-Bissau OR Liberia OR Mali OR Mauritania OR Niger OR Nigeria OR Senegal OR Sierra Leone OR Togo OR Xhosa OR Xosa OR Bantu OR Rundi OR Rwanda OR Shona OR Zulu OR Lingala OR Swahili OR Hausa OR Yoruba OR Ibo OR Igbo OR Kongo OR Fulani OR Amharic OR Oromo OR Chewa OR Sotho OR Showa OR Khoisan OR KhoiSan OR Khoi-San OR Khoe-San OR Malagasy OR Afrikaans OR Hausa OR Songhay OR Songhai OR Berber)  620,225  4  AB =("clinical trial"[pt] OR "random allocation" OR random* OR clinical trial OR clinical study OR controlled trial OR controlled study OR "Pilot Projects" OR ((phase i* OR phase 1* OR phase 2* OR phase 3*) AND (study OR studies OR trial)) OR program* OR intervention OR pilot* OR strateg*)  9,991,539  5  (#2) AND #3  50,910  6  #5 AND #1  4,220  7  #6 AND #4  3,247 | 3247 |
| CINAHL Plus with Full Text  EBSCO | ( ( ( (MH "Human Immunodeficiency Virus") OR (MH "HIV-Positive Persons") OR (MH "HIV Seropositivity") OR (MH "HIV Wasting Syndrome") OR (MH "HIV Seronegativity") OR (MH "HIV-AIDS Nursing") OR (MH "HIV Infections") OR (MH "HIV Education") OR (MH "HIV Enteropathy") ) AND AB ( ((Human AND Immunodeficiency AND Virus) OR (Acquired AND Immune AND Deficiency AND Syndrome) OR “Human Immunodeficiency Virus” OR “Acquired Immune Deficiency Syndrome Virus” OR AIDS OR AIDS Virus OR Seronegativities OR Seronegativity OR HIV OR hiv-1 OR hiv-2 OR hiv1 OR hiv2 OR "hiv infect*" OR “human immune deficiency virus” OR “human immuno-deficiency virus” OR “human immune-deficiency virus” OR “acquired immuno-deficiency syndrome” OR “acquired immune-deficiency syndrome” OR HIVAIDS OR HIV-positive OR HIV OR HIV-AIDS OR "HIV" OR "HIV Long-Term Survivors" OR "HIV Seronegativity" OR "HIV Infections" OR "HIV Seroprevalence" OR "HIV-2" OR "HIV-1" OR "HIV Seropositivity" OR HIV) ) AND ( (MH "Africa") OR (MH "Africa South of the Sahara") ) OR AB ( ("Africa" OR "Africa South of the Sahara" OR Africa* OR Subsaharan OR sub-saharan OR Cameroon OR Central African Republic OR Chad OR Congo OR Democratic Republic of the Congo OR Equatorial Guinea OR Gabon OR “Sao Tome and Principe” OR Burundi OR Djibouti OR Eritrea OR Ethiopia OR Kenya OR Rwanda OR Somalia OR South Sudan OR Sudan OR Tanzania OR Uganda OR Angola OR Botswana OR Eswatini OR Lesotho OR Malawi OR Mozambique OR Namibia OR South Africa OR Zambia OR Zimbabwe OR Benin OR Burkina Faso OR Cabo Verde OR “Cote d'Ivoire” OR Gambia OR Ghana OR Guinea OR Guinea-Bissau OR Liberia OR Mali OR Mauritania OR Niger OR Nigeria OR Senegal OR Sierra Leone OR Togo OR Xhosa OR Xosa OR Bantu OR Rundi OR Rwanda OR Shona OR Zulu OR Lingala OR Swahili OR Hausa OR Yoruba OR Ibo OR Igbo OR Kongo OR Fulani OR Amharic OR Oromo OR Chewa OR Sotho OR Showa OR Khoisan OR KhoiSan OR Khoi-San OR Khoe-San OR Malagasy OR Afrikaans OR Hausa OR Songhay OR Songhai OR Berber) ) ) AND ( ( (MH "Exercise") OR "exercise" OR (MH "Exercise Intensity") OR (MH "Exercise Test") OR (MH "Sport Specific Training") OR (MH "Exercise Test, Muscular") OR (MH "Group Exercise") OR (MH "Warm-Up Exercise") OR (MH "Exercise Test, Cardiopulmonary") OR (MH "Abdominal Exercises") OR (MH "Exercise Positions") OR (MH "Resistance Training") ) AND AB ( "Exercise" OR "Sports" OR "Physical Fitness" OR "Cardiorespiratory Fitness" OR "Movement" OR interval OR lifestyle* OR “physical activit*” OR “PA intervention*” OR endurance OR weight-lifting weight-bearing OR high-intensity OR HIIT OR cardiorespiratory OR cardio OR resistance OR strength OR athletic* OR working-out OR work-out OR strength-training OR sport* OR team OR steps or pedometer OR aerobic* OR muscle-strengthening OR bone-strengthening OR stretching OR exercise* ) ) ) AND ( ( (MH "Intervention Trials") OR (MH "Experimental Studies") OR "intervention" OR (MH "Clinical Trials") OR "clinical trials" OR (MH "Randomized Controlled Trials") OR (MH "Pilot Studies") OR (MH "Pilots") OR "pilot" OR (MH "Prospective Studies") OR (MH "Concurrent Prospective Studies") OR (MH "Historically Controlled Study") OR (MH "Nonexperimental Studies") OR (MH "Cross Sectional Studies") OR (MH "Study Design") OR (MH "Physiological Studies") OR (MH "Multimethod Studies") ) OR AB ( ("clinical trial"[pt] OR "random allocation" OR random* OR clinical trial OR clinical study OR controlled trial OR controlled study OR "Pilot Projects" OR ((phase i* OR phase 1* OR phase 2* OR phase 3*) AND (study OR studies OR trial)) OR program* OR intervention OR pilot* OR strateg*) ) ) | 1119 |
| African Index Medicus | ( collection_gim:("AIM") AND mh:("HIV" OR "Exercise" OR "Sports" OR "Physical Fitness")) | 637 |
