# Supplementary material for: Physical activity interventions for cardiometabolic and psychosocial outcomes in people living with HIV in sub Saharan Africa: A systematic review
Source: PLOS Glob Public Health. 2026 Sep 28;6(9):e0007070. doi: 10.1371/journal.pgph.0007070 (PMC13618956; doi:10.1371/journal.pgph.0007070)
Supplement: Supplementary file 3 [file pgph.0007070.s003.docx]

| **STUDY** | **Behavioural change techniques employed and their definitions** |
| --- | --- |
| **STUDY:** Mabweazara 2018  **Exercise modality:** Multimodal exercise  **Assessment of PA:** GPAQ; Pedometer | - Information about health consequences- Provide information (e.g., written, verbal, visual) about health consequences of performing the behaviour - Feedback on the behaviour- Monitor and provide informative or evaluative feedback on performance of the behaviour (e.g., form, frequency, duration, intensity) - Feedback on outcomes of the behaviour- Monitor and provide feedback on the outcome of performance of the behaviour - Goal-setting outcome- Set or agree on a goal defined in terms of a positive outcome of wanted behaviour - Self-monitoring of the behaviour- Establish a method for the person to monitor and record their behaviour(s) as part of a behaviour change strategy - Material incentive (behaviour)- Inform that money, vouchers or other valued objects will be delivered if and only if there has been effort and/or progress in performing the behaviour (includes ‘Positive reinforcement’) - Demonstration of the behaviour- Provide an observable sample of the performance of the behaviour, directly in person or indirectly e.g., via film, pictures, for the person to aspire to or imitate - Problem solving- Analyse , or prompt the person to analyse, factors influencing the behaviour and generate or select strategies that include overcoming barriers and/or increasing facilitators (includes ‘Relapse Prevention’ and ‘Coping Planning’) - Instruction on how to perform the behaviour- Advise or agree on how to perform the behaviour (includes ‘Skills training’) - Social support (unspecified)- Advise on, arrange, or provide social support (e.g., from friends, relatives, colleagues,’ buddies’ or staff) or noncontingent praise or reward for performance of the behaviour. It includes encouragement and counselling, but only when it is directed at the behaviour - Social support (practical)- Advise on, arrange, or provide practical help (e.g., from friends, relatives, colleagues, ‘buddies’ or staff) for performance of the behaviour - Goal-setting behaviour- Set or agree on a goal defined in terms of the behaviour to be achieved - Review the behavioural goals- Review behaviour goal(s) jointly with the person and consider modifying goal(s) or behaviour change strategy in light of achievement. This may lead to re-setting the same goal, a small change in that goal or setting a new goal instead of (or in addition to) the first, or no change - Action planning- Prompt detailed planning of performance of the behaviour (must include at least one of context, frequency, duration and intensity). Context may be environmental (physical or social) or internal (physical, emotional or cognitive) (includes ‘Implementation Intentions’) |
| **STUDY:** Roos 2014  **Exercise modality:** Aerobic exercise  **Assessment of PA:** Pedometer; Activity Diary. | - Information about health consequences- Provide information (e.g., written, verbal, visual) about health consequences of performing the behaviour - Feedback on the behaviour- Monitor and provide informative or evaluative feedback on performance of the behaviour (e.g., form, frequency, duration, intensity) - Goal-setting outcome- Set or agree on a goal defined in terms of a positive outcome of wanted behaviour - Self-monitoring of the behaviour- Establish a method for the person to monitor and record their behaviour(s) as part of a behaviour change strategy - Problem solving- Analyse , or prompt the person to analyse, factors influencing the behavior and generate or select strategies that include overcoming barriers and/or increasing facilitators (includes ‘Relapse Prevention’ and ‘Coping Planning’) - Instruction on how to perform the behaviour- Advise or agree on how to perform the behaviour (includes ‘Skills training’) |
