# Supplementary material for: Physical activity interventions for cardiometabolic and psychosocial outcomes in people living with HIV in sub Saharan Africa: A systematic review
Source: PLOS Glob Public Health. 2026 Sep 28;6(9):e0007070. doi: 10.1371/journal.pgph.0007070 (PMC13618956; doi:10.1371/journal.pgph.0007070)
Supplement: Supplementary file 5 [file pgph.0007070.s005.docx]

S3 Table. Articles after-full text review and reasons for exclusion

| **Authors** | **Title** | **Year** | **Journal** | **Decision** | **Reasons for exclusion** |
| --- | --- | --- | --- | --- | --- |
| 1. Aweto et al. | Effects of Aerobic Exercise on the Pulmonary Functions, Respiratory Symptoms and Psychological Status of People Living With HIV | 2016 | J Res Health Sci | Included |  |
| 2. Daniels & Van Niekerk | The impact of a therapeutic exercise intervention on depression and body self-image in HIV-positive women in sub-Saharan Africa | 2018 | HIV | Included |  |
| 3. Ezema et al. | Effect of aerobic exercise training on cardiovascular parameters and CD4 cell count of people living with human immunodeficiency virus/acquired immune deficiency syndrome: A randomized controlled trial | 2014 | Niger J Clin Pract | Included |  |
| 4. Ijego et al. | Assessment of Progressive Resistance Exercise Training on CD4 Count and Weight of People with HIV/AIDS in A University Teaching Hospital, Ebonyi State Nigeria | 2014 | Biosc Biotech Res Comm | Included |  |
| 5. John et al | Effects of a 6-week aerobic exercise programme on the cardiovascular parameters, body composition, and quality of life of people living with human immune virus | 2018 | J Exerc Rehabil | Included |  |
| 6. Mabweazara et al. | A six week contextualised physical activity intervention for women living with HIV and AIDS of low socioeconomic status: a pilot study | 2018 | AIDS Care | Included |  |
| 7. Maduagwu et al. | Aerobic Exercise Improves Quality of Life and CD4 Cell Counts in HIV Seropositives in Nigeria | 2017 | JHVRV | Included |  |
| 8. Maharaj et al. | Rehabilitation program for the quality of life for individuals on highly active antiretroviral therapy in KwaZulu-Natal, South Africa: a short report | 2011 | International Journal of Rehabilitation Research | Included |  |
| 9. Mangona et al. | Effect of Different Types of Exercise in HIV + Mozambican Women Using Antiretroviral Therapy | 2015 | TOAIDJ | Included |  |
| 10. Mbayo & Sookan | Effects of a resistance training programme in people living with HIV in Zimbabwe | 2020 | Sport Sci Health | Included |  |
| 11. Mkandla et al. | The effects of progressive-resisted exercises on muscle strength and health-related quality of life in persons with HIV-related poly-neuropathy in Zimbabwe | 2016 | AIDS Care | Included |  |
| 12. Mutimura et al. | Exercise Training Reduces Central Adiposity and Improves Metabolic Indices in HAART-Treated HIV-Positive Subjects in Rwanda: A Randomized Controlled Trial | 2008 | AIDS Research and Human Retroviruses | Included |  |
| 13. Mutimura et al. | The effects of exercise training on quality of life in HAART-treated HIV-positive Rwandan subjects with body fat redistribution | 2008 | Qual Life Res | Included |  |
| 14. Odunaiya et al. | Effects of a four-week aerobic exercise programme on depression, anxiety and general self-efficacy in people living with HIV on highly active anti-retroviral therapy | 2022 | AIDS Care | Included |  |
| 15. Roos et al. | Effects of an Education and Home-Based Pedometer Walking Program on Ischemic Heart Disease Risk Factors in People Infected with HIV: A Randomized Trial | 2014 | JAIDS | Included |  |
| 16. Shittu et al. | Effect of aerobic exercise on functional capacity and health status of individuals living with HIV/AIDS in Kano, Nigeria: pilot study | 2020 | Physiotherapy | Included |  |
| 17. Maduagwu et al. | Effect of Aerobic Exercise on CD4 Cell Count and Lipid Profile of HIV Infected Persons in North Eastern Nigeria | 2015 | J AIDS Clin Res | Included |  |
| 18. Tumusiime et al. | The effects of a physiotherapist-led exercise intervention on peripheral neuropathy among people living with HIV on antiretroviral therapy in Kigali, Rwanda | 2019 | South African Journal of Physiotherapy |  | Did not assess any of our pre-specified outcomes of interest, |
